# Supplementary material for: Effectiveness of cash-plus programmes on early childhood outcomes compared to cash transfers alone: A systematic review and meta-analysis in low- and middle-income countries
Source: PLoS Med. 2021 Sep 28;18(9):e1003698. doi: 10.1371/journal.pmed.1003698 (PMC8478252; doi:10.1371/journal.pmed.1003698)
Supplement: S1 Checklist — PRISMA, Preferred Reporting Items for Systematic Reviews and Meta-Analyses. (DOC) [file pmed.1003698.s001.doc]

| **Section/topic** | **#** | **Checklist item** | **Reported on page #** |
| --- | --- | --- | --- |
| **TITLE** | | |  |
| Title | 1 | Identify the report as a systematic review, meta-analysis, or both.  ***Evidence***: Revised title, “Effectiveness of cash-plus programmes on early childhood outcomes compared to cash transfers alone: A systematic review and meta-analysis in low- and middle-income countries.” | Title Page |
| **ABSTRACT** | | |  |
| Structured summary | 2 | Provide a structured summary including, as applicable: background; objectives; data sources; study eligibility criteria, participants, and interventions; study appraisal and synthesis methods; results; limitations; conclusions and implications of key findings; systematic review registration number.  ***Evidence***: All categories are presented in the abstract. | Abstract |
| **INTRODUCTION** | | |  |
| Rationale | 3 | Describe the rationale for the review in the context of what is already known.  ***Evidence Excerpt***: “[Cash transfer] impacts have been more mixed and less overwhelming in more challenging areas of child development, including nutrition [4] and health [5]. In efforts to strengthen the impact of these interventions, cash transfers have begun to be packaged as cash-plus programmes, combining cash transfers with other interventions or services… Cash-plus interventions hold much promise in accelerating achievement of multiple SDG targets for children. However, to the best of our knowledge to date, there has been no synthesis to evaluate if these multisectoral interventions are more effective than cash transfers alone.” | Introduction paragraph 1-4 |
| Objectives | 4 | Provide an explicit statement of questions being addressed with reference to participants, interventions, comparisons, outcomes, and study design (PICOS).  ***Evidence***: “*Are cash-plus interventions for infants and children under the age of 5 more effective than cash transfers alone in improving child health and wellbeing outcomes across the SDGs?”* | Introduction paragraph 5 |
| **METHODS** | | |  |
| Protocol and registration | 5 | Indicate if a review protocol exists, if and where it can be accessed (e.g., Web address), and, if available, provide registration information including registration number.  ***Evidence***: “The protocol was pre-registered with PROSPERO (CRD42018108017).” | Methodology Paragraph 1 |
| Eligibility criteria | 6 | Specify study characteristics (e.g., PICOS, length of follow-up) and report characteristics (e.g., years considered, language, publication status) used as criteria for eligibility, giving rationale.  ***Evidence Summary***: Intervention package implemented in LMICs, targeted to families with children <5 and contain a cash transfer and at least one plus component focused on nutrition, health, education, or violence prevention. Outcomes included measures of poverty, malnutrition, mortality, morbidity from infectious disease, child development, and violence against children. Studies had to have a cash only arm and a cash-plus arm and be conducted from 2000-2021, using an experimental or quasi-experimental design. Full criteria and explanations listed in main paper. | Methodology Eligibility Criteria Paragraph 1-9 |
| Information sources | 7 | Describe all information sources (e.g., databases with dates of coverage, contact with study authors to identify additional studies) in the search and date last searched.  ***Evidence Summary***: “We searched eleven electronic databases, twenty-seven grey literature sources, and four trial registries. Four journals were hand-searched and ten experts in the field of development economics and cash-plus interventions were contacted to identify unpublished literature. The information sources and sample search strategy are listed in S1 Tables. All searches were completed through 06 April 2021.” | Methodology Information Sources Paragraph 1, S1 |
| Search | 8 | Present full electronic search strategy for at least one database, including any limits used, such that it could be repeated.  ***Evidence***: “The search strategy was informed by literature on the review topic and contains categories for the regions and individual countries, the population (infants and children), and terms for social protection interventions, which were replicated and modified from Owusu-Addo and colleagues (2016)…. The information sources and sample search strategy are listed in S1 Tables.” | Methodology Information Sources Paragraph 1, S1 |
| Study selection | 9 | State the process for selecting studies (i.e., screening, eligibility, included in systematic review, and, if applicable, included in the meta-analysis).  ***Evidence***: “Studies were imported into Rayyan and de-duplicated prior to screening [15]. Studies were double-screened blind to reduce potential bias. Initial agreement between raters was > 96% and reviewers agreed on the final included studies…. Studies were meta-analysed when there were at least three studies for an outcome with similar follow up times for the same cash-plus intervention.” | Methodology Study Selection Paragraph 1, Data Synthesis Paragraph 1 |
| Data collection process | 10 | Describe method of data extraction from reports (e.g., piloted forms, independently, in duplicate) and any processes for obtaining and confirming data from investigators.  ***Evidence***: “A standardised data extraction form was used, with categories for population characteristics and context, intervention design and components, outcomes and effects, pathways and equity evidence [16-19]. Two authors extracted meta-analysis data independently to minimise errors.” | Methodology Study Selection Paragraph 1 |
| Data items | 11 | List and define all variables for which data were sought (e.g., PICOS, funding sources) and any assumptions and simplifications made.  ***Evidence Summary***: “A standardised data extraction form was used, with categories for population characteristics and context, intervention design and components, outcomes and effects, pathways and equity evidence [16-19].” Outcomes considered in review “include measures of poverty (including multidimensional poverty), malnutrition (including stunting, wasting, underweight, and obesity), morbidity or mortality (neonatal, infant, and children under 5) including from unsafe water or lack of sanitation/hygiene or infectious disease, psychosocial and cognitive development, and violence against children.” | Methodology Study Selection Paragraph 1, Eligibility Criteria Paragraph 6, Table 1 |
| Risk of bias in individual studies | 12 | Describe methods used for assessing risk of bias of individual studies (including specification of whether this was done at the study or outcome level), and how this information is to be used in any data synthesis.  ***Evidence:*** “Assessing for risk of bias at the study level, randomised studies were evaluated using the Cochrane Risk of Bias Tool and non-randomised studies using the ROBINS-I tool [20,21]. All studies were double coded, and disagreements discussed. Piloting of the risk-of-bias assessment on six studies had 100% agreement between raters and > 95% for the full set of studies. Visualisations were created using *robvis* software [22]*.* The quality assessment was used to comment on the strength and limitations of the evidence-base and the confidence in recommendations from the synthesis [23]. Due to a limited number of retrieved studies across a range of cash-plus combinations and outcomes, no meaningful analysis could be conducted to assess for publication bias.” | Methodology Risk of Bias Paragraph 1 |
| Summary measures | 13 | State the principal summary measures (e.g., risk ratio, difference in means).  ***Evidence***: “Standardised mean differences (d) for continuous outcomes (i.e., cognitive development and anthropometric z-scores) and log odds ratios for binary outcomes (i.e., anthropometric outcome, violence against children, and positive parenting)” | Methodology Data Synthesis Paragraph 2 |
| Synthesis of results | 14 | Describe the methods of handling data and combining results of studies, if done, including measures of consistency (e.g., I^2^) for each meta-analysis.  ***Evidence Summary***: “Studies were meta-analysed when there were at least three studies for an outcome with similar follow-up times for the same cash-plus intervention… In instances when a study used a pure (no-intervention) control with two treatment arms (cash-only and cash-plus), results were re-analysed using the cash-only arm as the control. The analysis corrected for clustering to address possible unit of analysis error by adjusting the sample size as necessary [24]…. Random-effects meta-analyses [were done] in R using the Knapp & Hartung adjustment, which accounts for few data points [26-28]. Quantitative measures of consistency/heterogeneity are reported (*I^2^* and τ*^2^*). Confidence intervals for I2 are also provided for context on the uncertainty of the value [29].” | Methodology Data Synthesis Paragraph 1-3 |

| Risk of bias across studies | 15 | Specify any assessment of risk of bias that may affect the cumulative evidence (e.g., publication bias, selective reporting within studies).  ***Evidence***: “Due to a limited number of retrieved studies across a range of cash-plus combinations and outcomes, no meaningful analysis could be conducted to assess for publication bias.” Cochrane Risk of Bias assessment includes a domain for incomplete reporting (noted in Section 12 above). | Methodology Risk of Bias Paragraph 1 |
| --- | --- | --- | --- |
| Additional analyses | 16 | Describe methods of additional analyses (e.g., sensitivity or subgroup analyses, meta-regression), if done, indicating which were pre-specified.  ***Evidence***: “Study effects were synthesised narratively when there were too few studies to meta-analyse. When studies were narratively synthesised, effect sizes were transformed to cash-only vs cash plus comparisons when possible. Intervention design was classified by four criteria set forth in a previous review of Cash + Parenting Programmes [30]. Quantitative equity effects in impact were noted, defined as either sub-group analyses or interaction effects [31].” | Methodology Data Synthesis Paragraph 3 |
| **RESULTS** | | |  |
| Study selection | 17 | Give numbers of studies screened, assessed for eligibility, and included in the review, with reasons for exclusions at each stage, ideally with a flow diagram.  ***Evidence***: “From 5097 unique articles identified in the search, 80 full-text articles were reviewed for inclusion. Sixty-three were excluded (see flow chart below and S2 Table); the majority were excluded either because the study did not have a cash-only group, or the study did not meet the criteria for a cash-plus programme. Eleven protocols were identified that could meet the criteria for inclusion in an update to the review; study teams were contacted, who all confirmed no intervention impact data was yet available. Seventeen studies were included in the review, of which eleven were meta-analysed (Figure 1 & S2 Table). The studies included in this review represent eleven unique cash-plus programmes. | Results Study selection Paragraph 1, Fig 1, S2 |
| Study characteristics | 18 | For each study, present characteristics for which data were extracted (e.g., study size, PICOS, follow-up period) and provide the citations.  ***Evidence***: Table 1 includes cash transfer, intervention designs, type of plus component, and outcomes measured. Further information (cash values, programme details and intensity/providers), follow-up period, study design, etc. are available in S3 Table. | Results Study Characteristics Paragraph 1-6, Table 1, S3 |
| Risk of bias within studies | 19 | Present data on risk of bias of each study and, if available, any outcome level assessment (see item 12).  ***Evidence***: “Risk of bias was generally low for most studies, emphasising a high-quality evidence base and strong methodological rigour. One study was rated as having some concerns in risk of bias because of deviations from the planned intervention, specifically in implementation challenges that led to difficulties in individuals accessing cash [44]. All randomised trials followed children longitudinally throughout the trial period. The three quasi-experimental studies were rated as having moderate risk of bias because although the studies account for confounding, there is still greater risk of bias than if the studies could have used a randomise design. Similarly, these three studies relied on repeat cross-sectional data, which introduces some bias in potentially uneven exposure to the intervention by assessing within-population change rather than within-person change. No study was rated as having high risk of bias. Individual study assessments are available in S4 Figures.” | Results Risk of Bias of Individual Studies Paragraph 1, S4 |
| Results of individual studies | 20 | For all outcomes considered (benefits or harms), present, for each study: (a) simple summary data for each intervention group (b) effect estimates and confidence intervals, ideally with a forest plot.  ***Evidence***: Table 2 gives summary of findings by intervention type, outcome, and comparison. Fig 2-4 present forest plots, including effect sizes, confidence intervals, and p-values. | Table 2, Fig 2-7 |
| Synthesis of results | 21 | Present results of each meta-analysis done, including confidence intervals and measures of consistency.  ***Evidence:*** Cash + Nutrition BCC (Fig 2-3): no added impact on z-score measures of anthropometrics for stunting (d= 0.03 (-0.04, 0.09), p= 0.36, I^2^= 24% (0, 88)), wasting (d= -0.03 (-0.12, 0.07), p= 0.51, I^2^= 47% (0, 93)), or being underweight (d= -0.03, (-0.11, 0.05), p= 0.45, I^2^= 31% (0, 92)). No added impact in reducing odds of children being stunted (OR= 0.95 (95%CI 0.83, 1.09), p= 0.40; I^2^= 26% (95%CI 0, 87)), wasted (OR= 0.99 (0.93, 1.05), p= 0.64; I^2^= 0% (0, 14)), or underweight (OR= 1.01 (0.84, 1.20), p= 0.93; I^2^= 17% (0, 98)).  Cash + Food (Fig 4-5): Added impact in increasing height-for-age z-score (d= 0.08 (0.03, 0.14), p= 0.02, I^2^=0% (0, 74)), which translated to significantly reduced odds of children being stunted (OR= 0.82 (0.74, 0.92), p= 0.01, I^2^= 0% (0, 70)). There was no added impact in improving weight-for-height z-score (d= -0.13 (-0.42, 0.16), p= 0.24, I^2^= 87% (57, 99)) or weight-for-age z-score (d= -0.06 (-0.28, 0.15), p= 0.43, I^2^= 76% (24, 98)). No added impact in reducing odds of children experiencing wasting (OR= 0.89 (0.70, 1.14), p= 0.24, I^2^= 0% (0, 86)) or underweight status (OR= 0.93, (0.80, 1.09), p= 0.26, I^2^= 0% (0, 84)), respectively.  Cash + Psychosocial Stimulation (Fig 6): may not be more effective than cash transfers alone in promoting overall cognitive development (d= 0.16 (-0.25, 0.57), p= 0.24, I^2^= 85% (47, 100)), although there is substantial heterogeneity among the studies.  Cash + Child Protection (Fig 7): is not more effective than cash alone in reducing parental violent discipline of children (OR= 0.83 (0.59, 1.17), p= 0.15, I^2^= 40% (0, 99)) or increasing exclusive use of positive parenting (OR= 1.02 (0.82, 1.28), p= 0.69, I^2^= 17% (0, 96)) | Results Synthesis of Results, Fig 2-7 |
| Risk of bias across studies | 22 | Present results of any assessment of risk of bias across studies (see Item 15).  ***Evidence:*** As noted in the methodology, “Due to a limited number of retrieved studies across a range of cash-plus combinations and outcomes, no meaningful analysis could be conducted to assess for publication bias.” Cochrane Risk of Bias domain for incomplete reporting in individual studies is reported in S4 Figures. | Methodology Risk of Bias Paragraph 1, S4 |
| Additional analysis | 23 | Give results of additional analyses, if done (e.g., sensitivity or subgroup analyses, meta-regression [see Item 16]).  ***Evidence***: Table 2 gives an overview map of findings, including from studies that could not be meta-analysed. In addition to the meta-analysis results, the narrative synthesis findings suggest that Cash + Primary Healthcare may lead to greater reductions in child mortality than cash alone, Cash + Child Protection in reducing violent discipline, and Cash + Nutrition BCC in reducing long-term poverty. Analysis is also included on the impact of intermediary outcomes in the pathway between cash-plus to child outcomes.  On Cash + Nutrition BCC: “Sensitivity analyses were also conducted by removing the quasi-experimental study [46] from the meta-analyses. No sensitivity test showed a significant z-score change for height-for-age (d= 0.04 (-0.03, 0.11), p= 0.18), weight-for-height (d= -0.03 (-0.15, 0.09), p= 0.57), or weight-for-age (d= -0.02 (-0.13, 0.08), p= 0.61).” | Results Synthesis of Results |
| **DISCUSSION** | | |  |
| Summary of evidence | 24 | Summarize the main findings including the strength of evidence for each main outcome; consider their relevance to key groups (e.g., healthcare providers, users, and policy makers).  ***Evidence***: “Meta-analysis results concluded that Cash + Food Transfers may be more effective than cash alone in reducing stunting. However, meta-analysis findings suggest no added impact above cash alone in Cash + Nutrition BCC for reducing undernutrition, Cash + Psychosocial Stimulation for improving cognitive development, or Cash + Child Protection for reducing violent discipline or increasing exclusive positive parenting. However, there was potentially substantial heterogeneity across the meta-analyses. The narrative synthesis found preliminary evidence that Cash + Primary Healthcare may have greater impacts than cash alone in reducing mortality, Cash + Food Transfers may have greater impacts than cash alone in reducing acute malnutrition in crisis contexts, Cash + Nutrition BCC may have greater impacts than cash alone in reducing poverty, and Cash + Child Protection trials suggest a trend toward greater impact than cash alone in reducing violent discipline. ” | Discussion Summary of evidence Paragraph 3 |
| Limitations | 25 | Discuss limitations at study and outcome level (e.g., risk of bias), and at review-level (e.g., incomplete retrieval of identified research, reporting bias).  ***Evidence Summary:*** Key limitations included few study numbers retrieved for each cash-plus programme, potentially high heterogeneity across studies in meta-analyses, only examining studies in English. | Discussion Limitations Paragraph 1-4 |
| Conclusions | 26 | Provide a general interpretation of the results in the context of other evidence, and implications for future research.  ***Evidence Summary***: The discussion includes interpretation of evidence in context to other systematic reviews on the effectiveness of cash transfers (generally) and on undernutrition specifically. There is also discussion on possible gender implications of the programming and its similarities to graduation programmes for early child development.  Summary of Implications for Future Research: There is discussion of the ideal study design, the need for more studies in evaluating if these programmes are more effective than cash alone, whether these programmes have long-term impacts and whether the effects are maintained at scale. Additional research is needed in optimising different design features. | Discussion Summary of Evidence Paragraph 1-7, Recommendations for Research Paragraph 1-5 |
| **FUNDING** | | |  |
| Funding | 27 | Describe sources of funding for the systematic review and other support (e.g., supply of data); role of funders for the systematic review.  ***Evidence***: “The authors received no specific funding for this work.” | [See PLOS Funding Statement] |

*From:*  Moher D, Liberati A, Tetzlaff J, Altman DG, The PRISMA Group (2009). Preferred Reporting Items for Systematic Reviews and Meta-Analyses: The PRISMA Statement. PLoS Med 6(6): e1000097. doi:10.1371/journal.pmed1000097
