# Supplementary figures and images for: Effectiveness of cash-plus programmes on early childhood outcomes compared to cash transfers alone: A systematic review and meta-analysis in low- and middle-income countries
Source: PLoS Med. 2021 Sep 28;18(9):e1003698. doi: 10.1371/journal.pmed.1003698 (PMC8478252; doi:10.1371/journal.pmed.1003698)

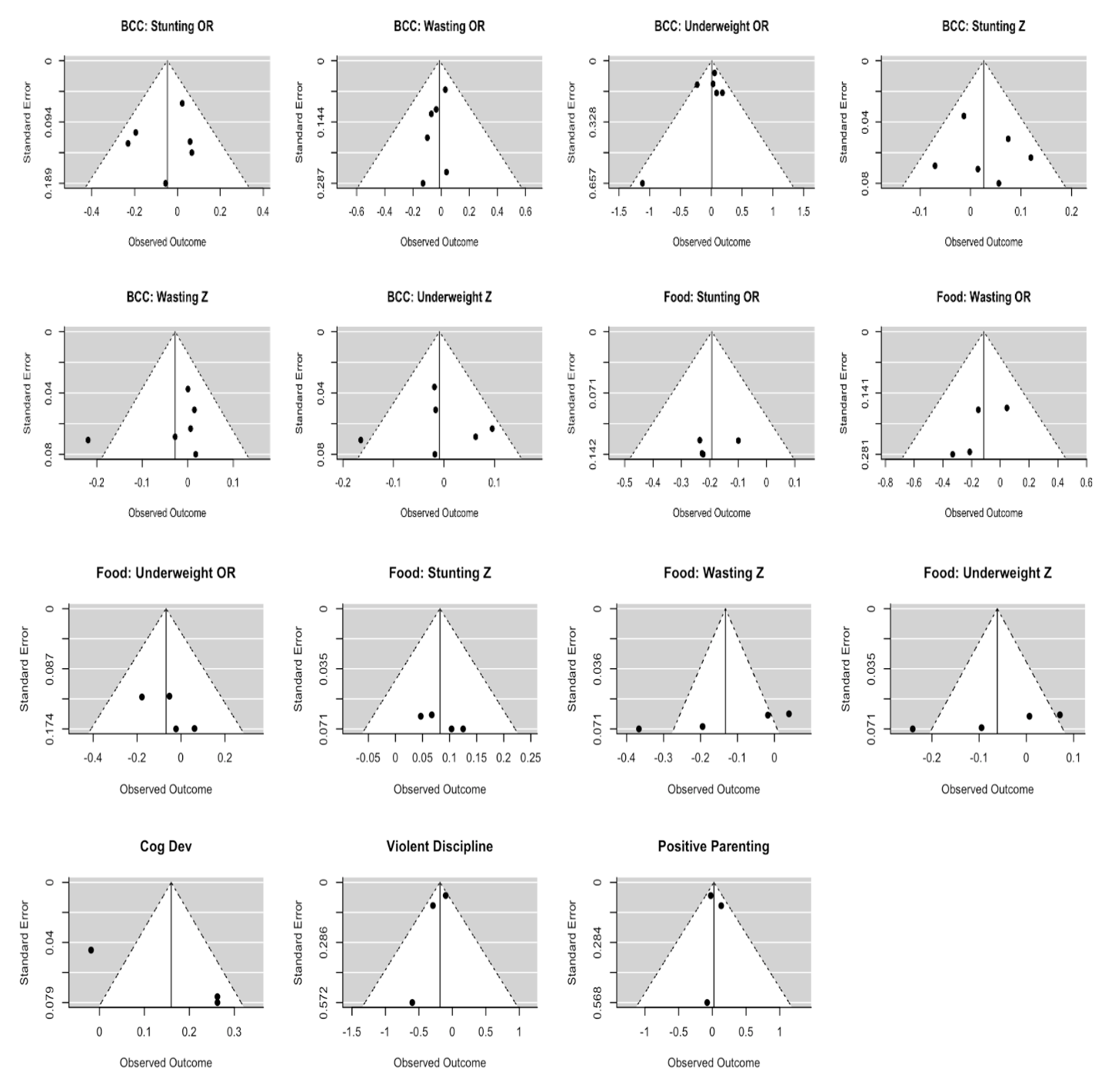

Supplement: S1 Fig — Funnel plots provided for each meta-analysis. (TIF) [file pmed.1003698.s002.tif]
