# Supplementary material for: Effectiveness of cash-plus programmes on early childhood outcomes compared to cash transfers alone: A systematic review and meta-analysis in low- and middle-income countries
Source: PLoS Med. 2021 Sep 28;18(9):e1003698. doi: 10.1371/journal.pmed.1003698 (PMC8478252; doi:10.1371/journal.pmed.1003698)
Supplement: S1 Table — Information provided on country, participants, follow-up period, study design, cash amount, intervention intensity, intervention provider, and plus-intervention descriptions for each study. (DOCX) [file pmed.1003698.s003.docx]

*S1 Table: Study Characteristics.*

| **Study** | **Country** | **Participants** | **Follow Up Period & Study Design** | **Cash Amount** | **Intervention Intensity** | **Intervention Provider** | **Plus-Intervention Description** |
| --- | --- | --- | --- | --- | --- | --- | --- |
| **Langendorf 2014** | Niger | Children 6-23 months | 4 months (Cluster RCT) | $52/month if also receiving food; $59 for cash alone. | Monthly measurement/ cash disbursements | Education assistant, community women, nutrition assistants/nurses | “Nutritious supplementary food, either HQ-LNS 500 kcal/day (Supplementary Plumpy, Nutriset), MQ-LNS 250 kcal/day (Plumpy’Doz, Nutriset), or Super Cereal Plus 820 kcal/day (Michiels and Cerfar).   BCC: Prior to each distribution, caregivers attended sessions, given information on topics such as breastfeeding, feeding children appropriately for their age group (e.g., enriched porridge and semi-solid snacks from 6 months of age then solid enriched foods from 12 months of age), food health and safety, prevention of malaria, vaccinations, and access to primary care based on Essential Nutrition Actions. Groups receiving cash transfer were given nutritional information on purchasing food for the targeted child, which included education on diet diversity (e.g., addition of enriched food such as oil, animal food, egg, beans, fruits, and vegetables to breast milk and local porridge)." |
| **Field 2021** | Myanmar | Pregnant women in 2nd trimester & Children 0-2 years | Endline measurement at age 24 months (Cluster RCT) | 10,000MMK/month (~$7.40), increased to 15,000MMK (~$11.09) in Oct 2017 | Monthly SBCC/cash disbursement | Save the Children partnered with Myanmar Nurses and Midwives Association and Pact Global Microfinance Fund. Delivered by Dept of Public Health in 40 villages in Pakokku. | BCC Activities in 4 categories (IYCF practices, health seeking behaviour, hygiene practices, household expenditures), done through different forums: • "Mother to Mother (m2m) Support Groups: covering Infant and Young Child Feeding (IYCF) and promoting uptake of maternal and child health care services etc. • Individual Counselling Services for mothers struggling with breastfeeding / complementary feeding (beginning Feb-18, at least 2 sessions for identified women) • Cooking demonstrations with mothers • Influential Caregiver Groups: behavioural change for husbands/grandmothers (beginning Jan-17, Men Edu Session; Elderly People Session) to influence & support MCH • Mobilisation of local authorities and health system" |
| **Khan 2019** | Pakistan | Children 6-23 months | 18 months (Cluster RCT) | 5000 PKR/quarter ($30/quarter, ~10/month) | Monthly measurements, quarterly community sessions and cash disbursement. | Used BISP (CT) national structure and provincial Integrated Reproductive Maternal Newborn & Child Health and Nutrition Program. Delivered by Lady Health Workers (LHWs). | Cash + Food: "Specialized Nutritious Food (SNF) is locally produced (Wawamum) made with heat treated (roasted) chickpeas, vegetable oils, dry skimmed milk, sugar, vitamins, minerals, emulsifier and antioxidants. A daily ration of 50-gram sachets was provided to cover the recommended daily allowance (RDA) of most micronutrients and 260 kcal of energy (about 1/4 of daily energy requirements) for children aged 6-23 months. Each recruited child received SNF for a duration of 18 months – from 6 months to 24 months of age.  Cash + SBCC: Health, nutrition and hygiene messages were provided by LHWs during routine monthly household visits. Community sessions were conducted quarterly with the help of a specialized picture-booklet by LHWs; Male & female group sessions arranged quarterly with help of health committees." |
| **Guyatt 2018** | Kenya | Pregnant women in 2nd trimester & Children 0-2 years | 16 months (RCT) | 2000KSH/month + 500-1000 KSH/month (~$24-29) depending on number of children and pregnant women in household. Control received 2000KSH/month (standard CT-OVC value, ~$19). | CHV visit monthly or bi-monthly, visits lasted ~2hrs | Community health volunteers (trained by PSK). | CHV made home visits, using material from the Ministry of Health maternal, infant and young child counselling card. Programme had 8 modules: "Maternal nutrition (iron/folic acid supplements and good water); Feeding infants 0-6 months (exclusive breastfeeding; Complementary feeding (6-23 months), vitamin A supplements; Feeding in special circumstances; Essential hygiene (WASH) actions, including ORS & Zinc; Growth monitoring and promotion; Developmental milestones; and Household food and nutrition security." The intervention also added text message and Beneficiary Learning Forums to re-enforce key messages. |
| **Ahmed 2019** | Bangladesh | Mothers living in poor, rural households with at least one child aged less than 2 years (0-24 months) at baseline | 2 years (Cluster RCT) | 1500 Taka/month ($19). | BCC weekly meeting | Community nutrition workers (CNWs). | Cash + Food: "monthly ration of 30 kg of rice, 2 kg of masoor pulse (lentil), and 2 litres of micronutrient-fortified cooking oil." Value of food was half the total transfer amount, the total of which equalled the amount of the cash-only group.BCC had 6 topics: "(1) importance of nutrition and diet diversity for health; (2) how handwashing and hygiene improve health; (3) diet diversity and micronutrients; (4) breastfeeding; (5) complementary foods for children 6-24 months; and (6) maternal nutrition. Taught through presentations, Q&A, interactive call and answer songs and chants, practical demonstrations, and role playing." Framed as soft condition for Cash + BCC group. CNWs made home visits to beneficiaries twice a month to follow up on topics discussed during BCC sessions and to discuss specific concerns mothers might have. CNWs conducted community meetings and met with influential members (village leaders, imams, elders) of villages in which BCC took place to explain purposes of nutrition training and to provide information being conveyed to study participants." |
| **Ahmed 2020** | Bangladesh | Mothers living in poor, rural households with at least one child aged less than 2 years (0-24 months) at baseline | 4 years post-intervention (Cluster RCT) | 1500 Taka/month ($19) | Follow up period after intervention ended. No intervention in 4-year period. | Follow up period after intervention ended. No intervention in 4-year period. | See above (Ahmed, 2019) |
| **UNICEF 2020** | Ethiopia | Children 6-23 months | 2 years (Quasi-Experimental) | Not reported. | BCC sessions bi-weekly | SWs, IN-SCT coordinators. SWs coordinated with Health Extension Workers (HEWs) and Development Agents (DAs). | "IN-SCT linked social workers (SWs) and community care coalitions (CCCs) with PSNP beneficiaries to strengthen access to health and nutrition services, complementary social services such as birth registration. In addition, developed BCC and gender and social development (GSD) materials with the community to develop capacity of CCCs and Women’s Development Army (WDAs).   Soft conditions (Schubert 2015): Attend four antenatal care visits & one postnatal; immunise children during first 9 months; growth monitoring monthly for 2 years, vitamin a/deworming; for children <5 suffering from acute malnutrition, follow guidelines for check-ups and supplementary and therapeutic feeding at local health clinics; attend monthly nutrition BCC sessions conducted by Health Extension Worker." |
| **Premand 2020** | Niger | Children 6-59 months | 2 years (Cluster RCT) | 10000FCFA/month ($20) | Cash monthly; BCC implemented through monthly village assemblies, community meetings and household visits (3 activities/month) | CT implemented by microfinance agencies and BCC by NGOs contracted by Safety Nets Unit. NGO field staff provide monthly village assembly and community educator deliver small-group meetings and home visits. | Each household participates in 3 activities/month: village assembly, small-group meeting, and home visit (soft conditions). BCC activities based on UNICEF Essential Family Practices (14 modules covering nutrition, health, psychosocial stimulation, and child protection). Used role play and theatre and visual aids. |
| **Barnhart 2020** | Rwanda | Children 6-36 months | 9 months (Cluster RCT) | Not reported. | Plus sessions 60-90 min/week for 3-4 months (home visits) | Community-based volunteers nominated by village chief. | Sugira Muryango (Strengthen the Family) is "strengths-based, home-visiting intervention adapted from Family Strengthening Intervention for HIV (FSI-HIV) and the WHO/UNICEF Care for Child Development Packages (2012). Intervention uses active coaching to promote responsive parenting, reduce family conflict, and increase caregivers’ abilities to access and navigate available resources. Rwandan songs/ proverbs were incorporated into curriculum to help internalise core skills. 12 modules targeting five key components: (1) Educating caregivers on children’s development, nutrition, health, and hygiene; (2) Coaching parents on responsive parenting and “serve and return” interactions; (3) Reducing violence by promoting family resilience in the face of adversity, positive parenting, and skills in conflict resolution; (4) Strengthening parental problem solving skills and social support through improved navigation of available formal and informal resources; and (5) Promoting early language learning and school readiness." (See Betancourt 2020 for details). |
| **Betancourt 2020** | Rwanda | Children 6-36 months | 9 months (Cluster RCT) | Not reported. | One 90 min module per week (~12 weeks) | Community based coaches (CBCs) | Families eligible for one of two versions of VUP programme, cash-for work programmes differing in labour intensity. "Modules: Family Narrative; importance of early stimulation and play; Building early communication skills; importance of good nutrition, hygiene and health; Managing stresses of parenting and family life; Resolving conflicts in the home; important role that everyone plays in raising a baby; Good parenting is better than being born well; Making the home a place where a baby’s brain can grow; With a united family, anything is possible. All visits included a 15-min “active play and communication” session where caregivers received live feedback on parent-child interactions." |
| **Jensen 2021** | Rwanda | Children 6-36 months | 18 months (1 year post- intervention) (Cluster RCT) | Not reported. | 1 booster visit at three- and six-months post-intervention for one hour each. | Community based coaches (CBCs) | In addition to the details described above (Barnhart, 2020; Betancourt, 2020), “coaches also helped families navigate formal resources such as government programmes to promote child health and nutrition including supplementation nutrition for malnourished children and informal support…. Three-month and six-month booster sessions occurred with the aim of each booster visit [to] reconnect with families, identify and address ongoing challenges, and engage caregivers in an ‘active play’ session modelled in each home-visiting session; each booster visit was approximately one hour.” |
| **Attanasio 2014** | Colombia | Children 12-24 months | 18 months (Cluster RCT) | Not reported. | Weekly change of toys | Locally elected representatives of FeA (Madres Líderes), and some supervision was provided by madre líder (mother leader). | Female community leader made weekly home visits, demonstrated activities with low-cost toys and books. Toys left at homes and changed weekly. Goal to enhance mother-child interactions and promote child development. Micronutrient supplementation Sprinkles (Hexagon Nutrition, Mumbai, India) were powdered, encapsulated micronutrients to be consumed daily for all children <6 to prevent/treat anaemia. CCT conditioned on preventive medical visits for children <5. Additional intervention details are available in the primary study’s data supplement. |
| **Andrew 2018** | Colombia | Children 12-24 months | 2 years post evaluation (Cluster RCT) | $8-16/month. | Weekly change of toys, nothing since intervention ended, cash continued | Locally elected representatives of FeA (Madres Líderes), and some supervision was provided by madre líder (mother leader). | Based on Jamaican home-visiting model available online under ‘Reach Up’ (see also Attanasio 2014) |
| **Fernald 2016** | Mexico | Children 0-18 months | 4 years (Cluster RCT) | Not reported. | Weekly meetings, 2 hours each (follows primary school calendar)  In one year, 26 sessions for mothers/fathers/care givers, 5 for fathers, 18 focusing on children, 8 for pregnant women, and 5 concluding sessions | Promotoras who are overseen by local supervisors, of who are overseen by program coordinators. | CT conditioned on attending ‘talleres’ (workshops on health topics) and preventive medical visits. Milk-based fortified food supplement was provided to households with pregnant/lactating women, children<2 or children under 5 with malnutrition.  EI designed to train rural-living parents whose children did not have access to pre-primary education. Sessions delivered in group setting (~20 women) at community centre weekly and covered hygiene, nutrition and stimulation activities to promote cognitive development. Weeks were targeted to different groups and toys were provided at sessions and parents encouraged to make some using household resources. |
| **Kagawa 2017** | Mexico | Children 0-18 months | 4 years (Cluster RCT) | See Fernald 2016 | See Fernald 2016 | See Fernald 2016 | See Fernald 2016 |
| **Guanais 2013** | Brazil | NA [Administrative Data] | 12 years (Quasi-Experimental) | Average $75.25/month | NA (Analysis of uneven scale up of CCT and primary healthcare) | Government, healthcare providers | CT conditioned on health checks for young children. Plus intervention was availability and provision of primary healthcare. |
| **da Silva 2019** | Brazil | NA [Administrative Data] | 6 years (Quasi-Experimental) | See Guanais 2013 for approximation. | NA (Analysis of uneven scale up of CCT and primary healthcare) | Government, healthcare providers | CT conditioned on health checks for young children. Plus intervention was availability and provision of primary healthcare. |
