# Supplementary material for: Effectiveness of cash-plus programmes on early childhood outcomes compared to cash transfers alone: A systematic review and meta-analysis in low- and middle-income countries
Source: PLoS Med. 2021 Sep 28;18(9):e1003698. doi: 10.1371/journal.pmed.1003698 (PMC8478252; doi:10.1371/journal.pmed.1003698)
Supplement: S1 Text — Sources of information searched and sample search strategy are provided. (DOCX) [file pmed.1003698.s004.docx]

*S1 Text: Information Sources & Sample Search Strategy.*

| Electronic Databases *(n=11)* | IBSS; ASSIA; Sociological Abstracts; Social Science Citation Index (Web of Science); Scopus; The Campbell Library: The Campbell Collaboration; PROQUEST Dissertations & Theses Global; Medline; PsycINFO; Global Health; Cochrane Public Health Group Specialised Register |
| --- | --- |
| Donor Agencies *(n=16)* | USAID Development Experience Clearinghouse; DFID (UK); UNICEF Evaluation Database; FAO; WHO Institutional Repository for Information Sharing; UNDP; World Bank Group Open Knowledge Repository; International Labour Organisation (ILO) LaborDoc Library; UNICEF-Innocenti Office of Research; African Development Bank; Asian Development Bank; Inter-American Development Bank; Save the Children; Partnership for Maternal, New-Born & Child Health; ECD Action Network; Innovations for Poverty Action |
| Trial Registries *(n=4)* | Clinical Trials (USA); Pan African Clinical Trials Registry; EU Clinical Trials Registry; American Economic Association RCT Registry |
| Other Grey Literature *(n=11)* | Google Search/Scholar; 3ie; British Library for Development Studies; Oxfam: Policy & Practice; UK Home Office Research Database; Transfer Project; Social Science Research Network; Centre for Social Protection (IDS, Sussex); socialprotection.org; Cash Learning Partnership Library; Oxford Policy Management. |
| Hand-searched Journals  *(n=4)* | The Lancet; Journal of Development Economics; Health Policy & Planning; Journal of Development Effectiveness |

*Sample search strategy (OVID).*

| 1 | Exp Developing Countries / |
| --- | --- |
| 2 | (Africa OR Central Africa OR Latin America OR Caribbean OR West Indies OR Eastern Europe OR Soviet OR South America OR Middle East OR low adj3 middle adj1 countr* OR LMIC OR LMICS OR LAMI Countr* OR Transitional countr*).ti,ab. |
| 3 | (AFGHANISTAN or ALBANIA or ALGERIA or SAMOA or ANGOLA or ARGENTINA or ARMENIA or AZERBAIJAN or BANGLADESH or BELARUS or BELIZE or BENIN or BHUTAN or BOLIVIA or BOSNIA or HERZEGOVINA or BOTSWANA or BRAZIL or BULGARIA or BURKINA FASO or BURUNDI or CABO VERDE or CAMBODIA or CAMEROON or CENTRAL AFRICAN REPUBLIC or CHAD or CHINA or COLOMBIA or COMOROS or CONGO or COSTA RICA or COTE adj1 IVOIRE or Ivory coast CUBA or DJIBOUTI or DOMINICA or DOMINICAN REPUBLIC or ECUADOR or EGYPT or SALVADOR or EQUATORIAL GUINEA or ERITREA or ETHIOPIA or FIJI or GABON or GAMBIA or GEORGIA or GHANA or GRENADA or GUATEMALA or GUINEA or GUINEA BISSAU or GUYANA or HAITI or HONDURAS or INDIA or INDONESIA or IRAN or IRAQ or JAMAICA or JORDAN or KAZAKHSTAN or KENYA or KIRIBATI or KOREA or KOSOVO or KYRGYZ or LAOS or LEBANON or LESOTHO or LIBERIA or LIBYA or MACEDONIA or MADAGASCAR or MALAWI or MALAYSIA or MALDIVES or MALI or MARSHALL ISLANDS or MAURITANIA or MAURITIUS or MEXICO or MICRONESIA or MOLDOVA or MONGOLIA or MONTENEGRO or MOROCCO or MOZAMBIQUE or MYANMAR or NAMIBIA or NEPAL or NICARAGUA or NIGER or NIGERIA or PAKISTAN or PANAMA or PAPUA NEW GUINEA or PARAGUAY or PERU or PHILIPPINES or ROMANIA or RUSSIA or RWANDA or SAO TOME adj1 PRINCIPE or SENEGAL or SERBIA or SIERRA LEONE or SOLOMON ISLANDS or SOMALIA or Somaliland or SOUTH AFRICA or SOUTH SUDAN or SRI LANKA or ST. LUCIA or ST. VINCENT or GRENADINES or SUDAN or SURINAME or SWAZILAND or SYRIA or TAJIKISTAN or TANZANIA or THAILAND or TIMOR LESTE or TOGO or TONGA or TUNISIA or TURKEY or TURKMENISTAN or TUVALU or UGANDA or UKRAINE or UZBEKISTAN or VANUATU or VENEZUELA or VIETNAM or WEST BANK or GAZA or YEMEN or ZAMBIA or ZIMBABWE).ti,ab. |
| 4 | 1 OR 2 OR 3 |
| 5 | MeSH: children OR infants / |
| 6 | (Child* OR kid* or boy* or girl* or baby or babies or toddler* or neonatal OR perinatal OR infant* OR infancy OR newborn OR pediatric OR paediatric).ti,ab. |
| 7 | 5 OR 6 |
| 8 | MeSH: Social welfare / |
| 9 | (((financial or cash or pay* or monetary or money or monies) adj2 (transfer* or measure* or incentive* or allowance* or exclu* or reform* or gain* or credit*1 or benefit*1)) or ((safety#net* or anti#poverty or poverty#averting or social#transfer or social#protection) adj2 (program* or intervention*)) or ((family or families) adj2 (polic* or program* or intervention*)) or (allowance* adj2 (intervention* or program* or assistance*)) or cash#plus or child support grant or economic incentive*).ti,ab. |
| 10 | 8 OR 9 |
| 11 | 4 AND 7 AND 10 |
|  | Limits: Years (2000-Current); English Language |
