# Supplementary material for: Effectiveness of cash-plus programmes on early childhood outcomes compared to cash transfers alone: A systematic review and meta-analysis in low- and middle-income countries
Source: PLoS Med. 2021 Sep 28;18(9):e1003698. doi: 10.1371/journal.pmed.1003698 (PMC8478252; doi:10.1371/journal.pmed.1003698)
Supplement: S2 Text — Table of excluded studies at full-text screening, with explanation, and a list of the studies included in the review. (DOCX) [file pmed.1003698.s005.docx]

*S2 Text: Excluded Studies at Full Text & List of Final Included Studies.*

| **Study (Author Year)** | **Reason for Exclusion** |
| --- | --- |
| ***Population*** | |
| [1] Chakrabarti 2020 | Intervention targeted to adolescents |
| [2] De Souza 2018 | No data on children < 5 |
| [3] Hoddinott 2017 | Non-cash transfer recipients |
| [4] Saville 2018 | Intervention for pregnancy & childbirth |
| ***Intervention*** | |
| [5] Beatty 2020 | No cash transfer |
| [6] Gelli 2019 | No cash transfer |
| [7] Banerjee 2010 | No cash transfer |
| [8] Lewis 2016 | Cash transfer does not meet criteria (once-off) |
| [9] Rosalina 2018 | No 'plus' intervention |
| [10] Akresh 2016 | No 'plus' intervention |
| [11] Robertson 2013 | No 'plus' intervention |
| [12] Reis 2010 | No 'plus' intervention |
| [13] Fernald 2008 | No 'plus' intervention |
| [14] de Souza 2021 | Plus intervention is environmental health intervention |
| [15] Daidone 2020 | Plus intervention is productivity/agriculture-based |
| [16] Bouguen 2019 | Plus intervention is productivity/agriculture-based |
| [17] Hurtado 2011 | Plus intervention is quality improvement of maternal & newborn care |
| [18] Morris 2004 | Plus intervention includes quality improvement of primary healthcare services |
| ***Comparison*** | |
| [19] Hidrobo 2020 | No cash-only group |
| [20] Kurdi 2020 | No cash-only group |
| [21] Adubra 2019 | No cash-only group |
| [22] Carneiro 2019 | No cash-only group |
| [23] Kurdi 2019 | No cash-only group |
| [24] Renzaho 2019 | No cash-only group |
| [25] Grijalva-Eternod 2018 | No cash-only group |
| [26] Raza 2018 | No cash-only group |
| [27] Renzaho 2018 | No cash-only group |
| [28] Zhang 2018 | No cash-only group |
| [29] OPM 2017 | No cash-only group |
| [30] Renzaho 2017 | No cash-only group |
| [31] Ramírez-Luzuriagac 2015 | No cash-only group |
| [32] Ramírez-Silva 2013 | No cash-only group |
| [33] Leroy 2008 | No cash-only group |
| [34] Isaurralde 2020 | No cash-only group, no baseline data |
| [35] Republic of Ghana 2018 | No designated cash-only group, all cash recipients offered plus-intervention |
| [36] Handa 2014 | No designated cash-only group, all cash recipients offered plus-intervention |
| [37] Park 2013 | No designated cash-only group, all cash recipients offered plus-intervention |
| [38] Sudfeld 2021 | Compares plus-only to cash-plus |
| [39] Briaux 2020 | Compares plus-only to cash-plus |
| [40] Fenn 2017 | Compares plus-only to cash-plus |
| [41] Grellety 2017 | Compares plus-only to cash-plus |
| [42] Levere 2016 | Compares plus-only to cash-plus |
| [43] Gilligan 2013 | Compares plus-only to cash-plus |
| ***Outcomes*** | |
| [44] Ahmed 2019 | No relevant outcomes for children < 5 |
| [45] Hoddinott, Ahmed, Karachiwalla 2018 | No third-order outcomes (only knowledge & practice) |
| [46] Hoddinott, Ahmed & Roy 2018 | No third-order outcomes (only nutrition knowledge) |
| ***Study Design*** | |
| [47] CARE 2020 | No control group |
| [48] Vieira-Meyer 2019 | Cross-sectional |
| [49] Huda 2018 | No control group |
| [50] Knauer 2016 | Pathway analysis |
| [51] Gilmour 2013 | Commentary |
| ***Protocol*** | |
| [52] Vaillant 2021 | No data available on intervention effectiveness |
| [53] Huda 2020 | No data available on intervention effectiveness |
| [54] Niescierenko 2020 | No data available on intervention effectiveness |
| [55] Seidenfeld 2020 | No data available on intervention effectiveness |
| [56] UNICEF-Innocenti 2020 | No data available on intervention effectiveness |
| [57] Gilligan 2019 | No data available on intervention effectiveness |
| [58] Hossain 2019 | No data available on intervention effectiveness |
| [59] UNICEF Madagascar 2017 | No data available on intervention effectiveness |
| [60] UNICEF Mauritania 2017 | No data available on intervention effectiveness |
| [61] Akram 2015 | No data available on intervention effectiveness |
| [62] de Walque 2015 | No data available on intervention effectiveness |
| ***Language*** | |
| [63] Neufeld 2013 | Only available in Spanish |

**Studies Excluded at Full-Text Screening**

1. Chakrabarti A, Handa S, Angeles G, Seidenfeld D. A cash plus program reduces youth exposure to physical violence in Zimbabwe. World Dev. 2020;134: 105037. doi:10.1016/j.worlddev.2020.105037

2. De Souza RA, Nery JS, Rasella D, Guimarães Pereira RA, Barreto ML, Rodrigues L, et al. Family health and conditional cash transfer in Brazil and its effect on tuberculosis mortality. Int J Tuberc Lung Dis. 2018;22: 1300–1306.

3. Hoddinott J, Ahmed I, Ahmed A, Roy S. Behavior change communication activities improve infant and young child nutrition knowledge and practice of neighboring non-participants in a cluster-randomized trial in rural Bangladesh. PLoS One. 2017;12: e0179866.

4. Saville NM, Shrestha BP, Style S, Harris-Fry H, Beard BJ, Sen A, et al. Impact on birth weight and child growth of participatory learning and action women’s groups with and without transfers of food or cash during pregnancy: Findings of the low birth weight South Asia cluster-randomised controlled trial (LBWSAT) in Nepal. PLoS One. 2018;13: e0194064.

5. Beatty A, Borkum E, Leith W, Henry M, Berends M, Null C, et al. MCC Indonesia Nutrition Project Impact Evaluation Final Report. Washington DC; 2020.

6. Gelli A, Gladstone M, Twalibu A, Nnensa T, Kariger P, Alderman H. Adding a nutrition behavior change communication component to an early childhood development intervention in Malawi: A cluster randomized trial. Washington, D.C.; 2019. Report No.: 01804.

7. Banerjee AV, Duflo E, Glennerster R, Kothari D. Improving immunisation coverage in rural India: Clustered randomised controlled evaluation of immunisation campaigns with and without incentives. BMJ. 2010;340: c2220.

8. Lewis A. Integrated food security programming and acute malnutrition prevention in the Central African Republic. F Exch. 2016.

9. Rosalina S, Sulaeman ES, Murti B. Effect of Family Hope Program on Maternal Health Behavior and Children Under Five Nutritional Status in Poor Families, Jombang, East Java. J Matern Child Heal. 2018;03: 33–43.

10. Akresh R, De Walque D, Kazianga H. Evidence from a randomized evaluation of the household welfare impacts of conditional and unconditional cash transfers given to mothers or fathers. Econ Work Pap Ser. 2016. Report No.: OKSWP11.

11. Robertson L, Mushati P, Eaton JW, Dumba L, Mavise G, Makoni J, et al. Effects of unconditional and conditional cash transfers on child health and development in Zimbabwe: A cluster-randomised trial. Lancet. 2013;381: 1283–1292.

12. Reis M. Cash transfer programs and child health in Brazil. Econ Lett. 2010;108: 22–25.

13. Fernald LC, Gertler PJ, Neufeld LM. Role of cash in conditional cash transfer programmes for child health, growth, and development: an analysis of Mexico’s Oportunidades. Lancet. 2008;371: 828–837.

14. De Souza AA, Mingoti SA, Paes-Sousa R, Heller L. Combined effects of conditional cash transfer program and environmental health interventions on diarrhea and malnutrition morbidity in children less than five years of age in Brazil, 2006-2016. PLoS One. 2021;16: 1–18. doi:10.1371/journal.pone.0248676

15. Daidone S, Pace N, Prifti E. Combining cash transfers with rural development interventions: Impact evaluation of Lesotho’s Child Grants Programme (CGP) and Sustainable Poverty Reduction through Income, Nutrition and access to Government Services (SPRINGS) project. Rome; 2020.

16. Bouguen A. Journal of Development Economics Registered Report Stage 1: The impact of a multidimensional program on nutrition and poverty in Burkina Faso. J Dev Econ. 2019.

17. Hurtado E, Richardson P, Broughton E. Analysis of Effectiveness and Cost-effectiveness of Adding Collaborative Improvement to a Conditional Cash Transfer Program in Guatemala. Res Eval Rep. Bethesda, MD; 2011.

18. Morris SS, Flores R, Olinto P, Medina JM. Monetary incentives in primary health care and effects on use and coverage of preventive health care interventions in rural Honduras: Cluster randomised trial. Lancet. 2004;364: 2030–2037.

19. Hidrobo M, Huybregts L, Karachiwalla N, Roy S. Multi-component cash transfer programs: Evidence from Mali’s social safety net program (Jigisémèjiri). 2020. Available: http://ebrary.ifpri.org/cdm/ref/collection/p15738coll2/id/133601

20. Kurdi S, Figueroa JL, Ibrahim H. Nutritional training in a humanitarian context: Evidence from a cluster randomized trial. Matern Child Nutr. 2020;16. doi:10.1111/mcn.12973

21. Adubra L, Le Port A, Kameli Y, Fortin S, Mahamadou T, Ruel MT, et al. Conditional cash transfer and/or lipid-based nutrient supplement targeting the first 1000 d of life increased attendance at preventive care services but did not improve linear growth in young children in rural Mali: Results of a cluster-randomized control. Am J Clin Nutr. 2019;110: 1476–1490.

22. Carneiro P, Kraftman L, Mason G, Moore L, Rasul I, Scott M. The Impacts of a Multifaceted Pre-natal Intervention on Human Capital Accumulation in Early Life. SSRN. 2019.

23. Kurdi S, Ghorpade Y, Ibrahim H. The Cash for Nutrition Intervention in Yemen: Impact Evaluation Study. MENA RP Work Pap. 2019. Report No.: 19.

24. Renzaho AMN, Chen W, Rijal S, Dahal P, Chikazaza IR, Dhakal T, et al. The impact of unconditional child cash grant on child malnutrition and its immediate and underlying causes in five districts of the Karnali Zone, Nepal - A trend analysis. Arch Public Heal. 2019;77: 1–18. doi:10.1186/s13690-019-0352-2

25. Grijalva-Eternod CS, Jelle M, Haghparast-Bidgoli H, Colbourn T, Golden K, King S, et al. A cash-based intervention and the risk of acute malnutrition in children aged 6–59 months living in internally displaced persons camps in Mogadishu, Somalia: A non-randomised cluster trial. PLoS Med. 2018;15: e1002684.

26. Raza WA, Van de Poel E, Van Ourti T. Impact and spill-over effects of an asset transfer program on child undernutrition: Evidence from a randomized control trial in Bangladesh. J Health Econ. 2018;62: 105–120.

27. Renzaho A, Chitekwe S, Chen W, Rijal S, Dhakal T, Chikazaza IR, et al. Impact of a multidimensional child cash grant programme on water, sanitation and hygiene in Nepal. J Water Sanit Hyg Dev. 2018;8: 520–532. doi:10.2166/washdev.2018.006

28. Zhang Y, Ji M, Zou J, Yuan T, Deng J, Yang L, et al. Effect of a conditional cash transfer program on nutritional knowledge and food practices among caregivers of 3–5-year-old left-behind children in the rural Hunan province. Int J Environ Res Public Health. 2018;15: 1–12.

29. Oxford Policy Management. Bihar Child Support Programme (BCSP): Impact Evaluation Endline Report. New Delhi; 2017.

30. Renzaho AMN, Chitekw S, Chen W, Rijal S, Dhakal T, Dahal P. The synergetic effect of cash transfers for families, child sensitive social protection programs, and capacity building for effective social protection on children’s nutritional status in Nepal. Int J Environ Res Public Health. 2017;14: 1–22. doi:10.3390/ijerph14121502

31. Ramírez-Luzuriagac MJ, Unar-Munguía M, Rodríguez-Ramírez S, Rivera JA, De Cosío TG. A food transfer program without a formal education component modifies complementary feeding practices in poor rural Mexican communities. J Nutr. 2016;146: 107–113.

32. Ramírez-Silva I, Rivera JA, Leroy JL, Neufeld LM. The Oportunidades program’s fortified food supplement, but not improvements in the home diet, increased the intake of key micronutrients in rural mexican children aged 12-59 months. J Nutr. 2013;143: 656–663.

33. Leroy JL, García-Guerra A, García R, Dominguez C, Rivera J, Neufeld LM. The Oportunidades program increases the linear growth of children enrolled at young ages in urban Mexico. J Nutr. 2008;138: 793–798.

34. Isaurralde M, Satriana S, Traoré AB, de Neubourg C. Summative Evaluation: Building Resilience for nutritional security in the Gambia through Social Transfers (BReST) 2016-2019. 2020. doi:10.31826/jlr-2017-151-201

35. Republic of Ghana. Ghana LEAP 1000 Programme: Endline Evaluation Report. 2018.

36. Handa S, Park M, Darko RO, Osei-Akoto I, Davis B, Daidone S. Livelihood Empowerment Against Poverty Program: Impact Evaluation. 2014.

37. Park MJ. Impact of social protection programs on child health and education in Ghana. UNC Chapel Hill Gillings School of Global Public Health. 2013.

38. Sudfeld CR, Bliznashka L, Ashery G, Yousafzai AK, Masanja H. Effect of a home-based health, nutrition, and responsive stimulation intervention and conditional cash transfers on child development and growth: a cluster-randomized controlled trial in Tanzania. medRxiv. 2021.

39. Briaux J, Martin-Prevel Y, Carles S, Fortin S, Kameli Y, Adubra L, et al. Evaluation of an unconditional cash transfer program targeting children’s first-1,000–days linear growth in rural Togo: A cluster-randomized controlled trial. PLoS Med. 2020;17: e1003388. doi:10.1371/journal.pmed.1003388

40. Fenn B, Colbourn T, Dolan C, Pietzsch S, Sangrasi M, Shoham J. Impact evaluation of different cash-based intervention modalities on child and maternal nutritional status in Sindh Province, Pakistan, at 6 mo and at 1 y: A cluster randomised controlled trial. PLoS Med. 2017;14: 1–24.

41. Grellety E, Babakazo P, Bangana A, Mwamba G, Lezama I, Zagre NM, et al. Effects of unconditional cash transfers on the outcome of treatment for severe acute malnutrition (SAM): A cluster-randomised trial in the Democratic Republic of the Congo. BMC Med. 2017;15: 1–19.

42. Levere M, Acharya G, Bharadwaj P. The Role of Information and Cash Transfers on Early Childhood Development: Evidence from Nepal. Natl Bur Econ Res. Washington DC; 2016. Report No.: 7931.

43. Gilligan D, Margolies A, Quinones E, Shalini R. Impact Evaluation of Cash and Food Transfers at Early Childhood Development Centers in Karamoja, Uganda: Final Impact Report. Washington DC; 2013.

44. Ahmed A, Hoddinott J, Roy S, Sraboni E. Transfers, Nutrition Programming, and Economic Well-being Experimental Evidence from Bangladesh. Washington DC; 2019. Report No.: 01879.

45. Hoddinott J, Ahmed A, Karachiwalla NI, Roy S. Nutrition behaviour change communication causes sustained effects on IYCN knowledge in two cluster-randomised trials in Bangladesh. Matern Child Nutr. 2018;14: e12498.

46. Hoddinott J, Ahmed A, Roy S. Randomized control trials demonstrate that nutrition-sensitive social protection interventions increase the use of multiple-micronutrient powders and iron supplements in rural pre-school Bangladeshi children. Public Health Nutr. 2018;21: 1753–1761.

47. CARE. Cash plus and nutrition outcomes. 2020.

48. Vieira-Meyer APGF, de Araújo Dias MS, Vasconcelos MIO, Rouberte ESC, de Almeida AMB, de Albuquerque Pinheiro TX, et al. What is the relative impact of primary health care quality and conditional cash transfer program in child mortality? Can J Public Heal. 2019;110: 756–767.

49. Huda TM, Alam A, Tahsina T, Hasan MM, Khan J, Rahman MM, et al. Mobile-based nutrition counseling and unconditional cash transfers for improving maternal and child nutrition in Bangladesh: Pilot study. JMIR mHealth uHealth. 2018;6: e156.

50. Knauer HA, Kagawa RMC, García-Guerra A, Schnaas L, Neufeld LM, Fernald LCH. Pathways to improved development for children living in poverty: A randomized effectiveness trial in rural Mexico. Int J Behav Dev. 2016;40: 492–499.

51. Gilmour S, Hamakawa T, Shibuya K. Cash-transfer programmes in developing countries. Lancet. 2013;381: 1254–1255.

52. Vaillant J, Goldstein M. Impact Evaluation of Cash Transfers and Social Promotion Activities in Mauritania. 2021.

53. Huda TM, Alam A, Tahsina T, Hasan MM, Iqbal A, Khan J, et al. Shonjibon cash and counselling: a community-based cluster randomised controlled trial to measure the effectiveness of unconditional cash transfers and mobile behaviour change communications to reduce child undernutrition in rural Bangladesh. BMC Public Health. 2020;20: 1–14. doi:10.1186/s12889-020-09780-5

54. Niescierenko M. Cash Transfers to Increase Dietary Diversity in Grand Gedeh County, Liberia. 2021.

55. Seidenfeld D, Bonilla J, Peterman A, Matine R, Tembe A, Handa S, et al. Impact Evaluation of the Child Grant 0-2 Programme in Mozambique Baseline Report. Washington DC; 2020.

56. UNICEF-Innocenti. Impact Evaluation of the Integrated Safety Net Programme in the Amhara Region of Ethiopia: Baseline Report. Florence, Italy; 2020.

57. Gilligan D, Ruel M, Hidrobo M, Roy S, Huybregts L. The Effect of a Cash Transfer Program and Preventive Nutrition Packages on Household Welfare and Child Nutritional Status in Mali (Jigisemejiri). 2019.

58. Hossain SJ, Roy BR, Salveen NE, Hasan MI, Tipu SMMU, Shiraji S, et al. Effects of adding psychosocial stimulation for children of lactating mothers using an unconditional cash transfer platform on neurocognitive behavior of children in rural Bangladesh: Protocol for a cluster randomized controlled trial. BMC Psychol. 2019;7: 1–6.

59. UNICEF Madagascar. Interventions dans le cadre du fonds additionnel du FSS nutrition et filets sociaux de securite dans le sud de Madagascar. Rapport d’analyse de l’Enquête Baseline FIAVOTA-2016. 2017.

60. UNICEF Mauritania. Programme Tekavoul Manuel de Mise en Œuvre. 2017.

61. Akram A, Khan A, Kurosaki T, Khalid H, Shonchoy A. An RCT to assess effectiveness of the in-home Growth Monitoring Tool (GroMoTo) in addressing chronic childhood undernutrition in Pakistan. 2015.

62. de Walque D, Kazianga H, Akresh R. Safety Nets and Early Childhood Development. 2015.

63. Neufeld LM, Guerra AG, Gaxiola ACF, Sánchez ADQ, Arenas AB, Cabrera AGH, et al. Evaluación de impacto del proyecto el Programa de Educación Inicial no Escolarizada de CONAFE (PEI-CONAFE) en sinergia con Oportunidades. [Impact evaluation of the CONAFE Non-School Early Education Program (PEI-CONAFE) project in synergy with Oportunidade. Cuernavaca, Mor., México; 2013.

**List of Included Studies:**

64. Ahmed A, Hidrobo M, Hoddinott J, Koch B, Roy S, Tauseef S. Social Protection and Sustainable Poverty Reduction: Experimental Evidence from Bangladesh. 2020. Report No.: 01988.

65. Ahmed A, Hoddinott J, Roy S. Food transfers, cash transfers, behavior change communication and child nutrition: Evidence from Bangladesh. 2019. Report No.: 01868.

66. Andrew A, Attanasio O, Fitzsimons E, Grantham-McGregor S, Meghir C, Rubio-Codina M. Impacts 2 years after a scalable early childhood development intervention to increase psychosocial stimulation in the home: A follow-up of a cluster randomised controlled trial in Colombia. PLoS Med. 2018;15.

67. Attanasio OP, Fernández C, Fitzsimons EOA, Grantham-McGregor SM, Meghir C, Rubio-Codina M. Using the infrastructure of a conditional cash transfer program to deliver a scalable integrated early child development program in Colombia: Cluster randomized controlled trial. BMJ. 2014;349: 1–12.

68. Barnhart DA, Farrar J, Murray SM, Brennan RT, Antonaccio CM, Sezibera V, et al. Lay-worker Delivered Home Visiting Promotes Early Childhood Development and Reduces Violence in Rwanda: A Randomized Pilot. J Child Fam Stud. 2020;29: 1804–1817.

69. Betancourt TS, Jensen SKG, Barnhart DA, Brennan RT, Murray SM, Yousafzai AK, et al. Promoting parent-child relationships and preventing violence via home-visiting: A pre-post cluster randomised trial among Rwandan families linked to social protection programmes. BMC Public Health. 2020;20: 1–11.

70. da Silva ES de A, Paes NA. Bolsa família programme and the reduction of child mortality in the municipalities of the Brazilian semiarid region. Cien Saude Colet. 2019;24: 623–630.

71. Fernald LCH, Kagawa RMC, Knauer HA, Schnaas L, Guerra AG, Neufeld LM. Promoting child development through group-based parent support within a cash transfer program: Experimental effects on children’s outcomes. Dev Psychol. 2016;53: 222–236.

72. Field E, Maffioli EM. Are Behavioral Change Interventions Needed to Make Cash Transfer Programs Work for Children? Experimental Evidence from Myanmar. NBER Work Pap Ser. Cambridge, MA; 2021. Report No.: 28443.

73. Guanais FC. The Combined Effects of the Expansion of Primary Health Care and Conditional Cash Transfers on Infant Mortality in Brazil, 1998–2010. Am J Public Health. 2013;103: 2000–2006.

74. Guyatt H, Klick M, Muiruri F, Rosa F Della. Final report : Evaluation of NICHE in the first 1,000 days of a child’s life in Kitui and Machakos counties, Kenya. Nairobi; 2018.

75. Jensen SK, Placencio-Castro M, Murray SM, Brennan RT, Goshev S, Farrar J, et al. Effect of a home-visiting parenting program to promote early childhood development and prevent violence: A cluster-randomized trial in Rwanda. BMJ Glob Heal. 2021;e003508. doi:10.1136/bmjgh-2020-003508

76. Kagawa RMC, Deardorff J, García-Guerra A, Knauer HA, Schnaas L, Neufeld LM, et al. Effects of a Parenting Program Among Women Who Began Childbearing as Adolescents and Young Adults. J Adolesc Heal. 2017;61: 634–641.

77. Khan GN, Kureishy S, Akbar N, Nasir M, Hussain M, Ahmed I, et al. A Stunting Prevention Cluster Randomized Controlled Trial: Leveraging the Social Protection System to Prevent Stunting in District Rahim Yar Khan, Punjab, Pakistan. Islamabad; 2019.

78. Langendorf C, Roederer T, de Pee S, Brown D, Doyon S, Mamaty AA, et al. Preventing Acute Malnutrition among Young Children in Crises: A Prospective Intervention Study in Niger. PLoS Med. 2014;11: e1001714.

79. Premand P, Barry O. Behavioral Change Promotion, Cash Transfers and Early Childhood Development: Experimental Evidence from a Government Program in a Low-Income Setting. Washington DC; 2020. Report No.: 9368.

80. UNICEF, MOLSA, IFPRI. Impact Evaluation of Improved Nutrition through Integrated Basic Social Services and Social Cash Transfer Pilot Program (IN-SCT) in Oromia and SNNP Regions, Ethiopia: Endline Impact Evaluation Report. 2020.
