# Supplementary material for: Effectiveness of cash-plus programmes on early childhood outcomes compared to cash transfers alone: A systematic review and meta-analysis in low- and middle-income countries
Source: PLoS Med. 2021 Sep 28;18(9):e1003698. doi: 10.1371/journal.pmed.1003698 (PMC8478252; doi:10.1371/journal.pmed.1003698)
Supplement: S3 Text — Individual study assessments for risk of bias using Cochrane Risk of Bias and ROBINS-I tools. (DOCX) [file pmed.1003698.s006.docx]

*S3 Text: Risk of Bias Assessments.*

*Cluster-Randomised Controlled Trials:*

**
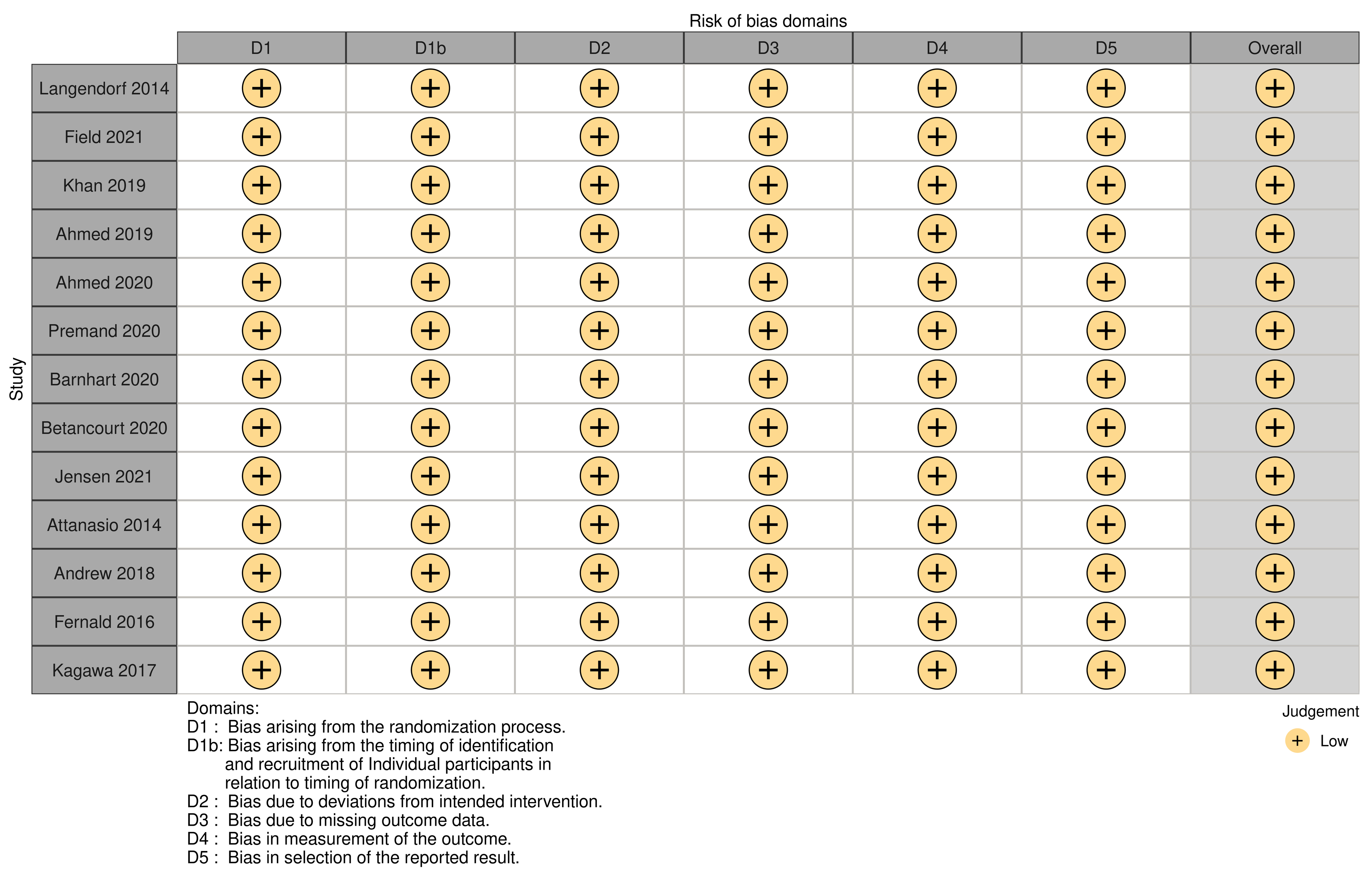
**

*Individual-Randomised Controlled Trial:*

*
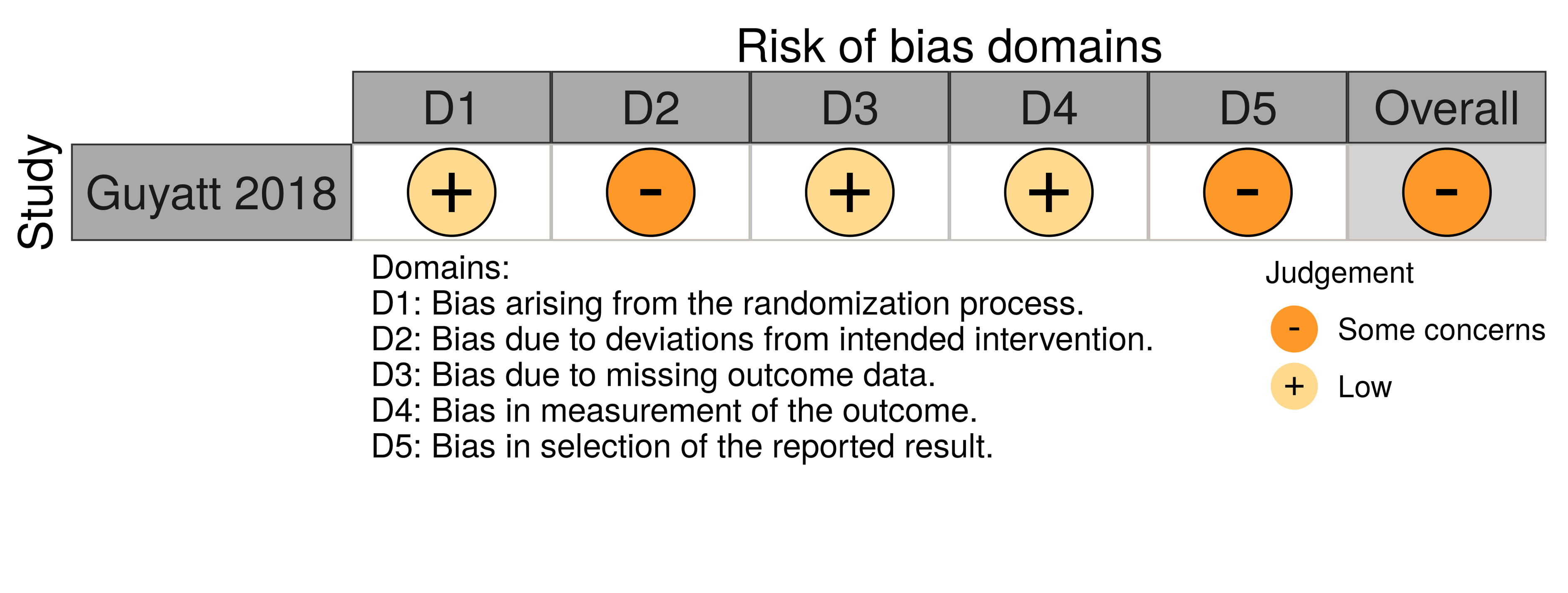
*

*ROBINS-I for Quasi-Experimental Studies:*

*
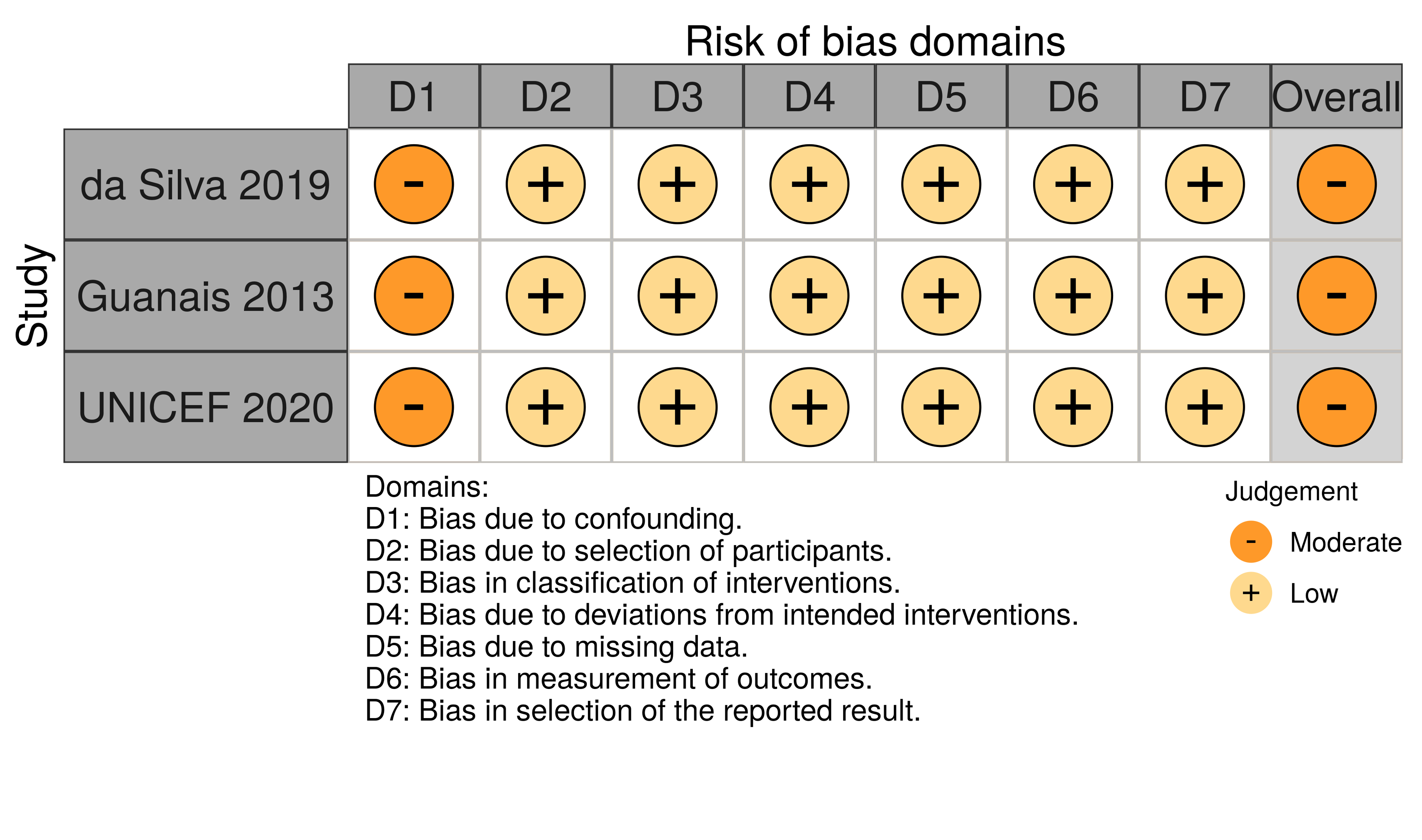
*
