# Supplementary material for: Effectiveness of cash-plus programmes on early childhood outcomes compared to cash transfers alone: A systematic review and meta-analysis in low- and middle-income countries
Source: PLoS Med. 2021 Sep 28;18(9):e1003698. doi: 10.1371/journal.pmed.1003698 (PMC8478252; doi:10.1371/journal.pmed.1003698)
Supplement: S4 Text — Study protocol as published on PROSPERO. (PDF) [file pmed.1003698.s007.pdf]

Implementation and evaluation of packaged cash-plus interventions to accelerate progress toward achieving the sdgs for infants and children in low- and middle-income countries: a systematic review and meta-analysis.

Madison Little, Keetie Roelen, Brittany CL Lange, Janina Steinert, Alexa Yakubovich, Lucie Cluver, David K Humphreys

Citation

Madison Little, Keetie Roelen, Brittany CL Lange, Janina Steinert, Alexa Yakubovich, Lucie Cluver, David K Humphreys. Implementation and evaluation of packaged cash-plus interventions to accelerate progress toward achieving the sdgs for infants and children in low- and middle-income countries: a systematic review and meta-analysis.. PROSPERO 2018 CRD42018108017 Available from: https://www.crd.york.ac.uk/prospero/display_record.php?ID=CRD42018108017

Review question

1. What packaged cash-plus interventions targeted to improve outcomes for infants and children under the age of 5 have been implemented and evaluated in low- and middle-income countries (LMICs)?

2. Are packaged cash-plus interventions effective in improving outcomes across the SDGs for children?

3. Do these interventions have an impact on equity (SDG10)?

4. Does conditionality affect the overall effectiveness of the package? Are there spillover or equity effects from conditionality?

5. What evaluation designs are used and do the evaluations consider the role of context and systems-level complexity? If so, what contextual factors influence the package effectiveness?

6. How are these programmes theorised to work and impact on SDG outcomes?

Searches

Electronic databases (n=11): IBSS; ASSIA; Sociological Abstracts; Social Science Citation Index (Web of Science); Scopus; The Campbell Library: The Campbell Collaboration; PROQUEST Dissertations & Theses Global; MEDLINE; PsycINFO; Global Health; Cochrane Public Health Group Specialised Register.

Donor agencies (n=16): USAID Development Experience Clearinghouse; DFID (UK); UNICEF Evaluation Database; FAO; WHO Institutional Repository for Information Sharing; UNDP; World Bank Group Open Knowledge Repository; International Labour Organisation (ILO) LaborDoc Library; UNICEF-Innocenti Office of Research; African Development Bank; Asian Development Bank; Inter-American Development Bank; Save the Children; Partnership for Maternal, New-Born & Child Health; ECD Action Network; Innovations for Poverty Action

Trial registries (n=4): Clinical Trials (USA); Pan African Clinical Trials Registry; EU Clinical Trials Registry; AEA RCT Registry.

Other grey literature (n=11): Google Search/Scholar; 3ie; British Library for Development Studies; Oxfam: Policy & Practice; Transfer Project; Social Science Research Network; Centre for Social Protection (IDS, Sussex); socialprotection.org; Cash Learning; Oxford Policy Management; ECD Action Network.

Hand-searched journals (n=4): The Lancet Global Health; Journal of Development Economics; Health Policy & Planning; Journal of Development Effectiveness.

Reference lists of final included studies will be examined to snowball potentially missed articles during the search. Experts and practitioners in the field will be contacted for any ongoing, unpublished trials that the

search would not have captured.

Studies conducted between 2000 and September 2020 in English.

Types of study to be included

Included studies must use an experimental or quasi-experimental design. Studies that do not measure the outcome at both baseline and post-intervention will be excluded. Because this review aims to evaluate potential interactions between cash and plus components, studies that evaluate no treatment (i.e. no cash or plus component) versus cash plus only will be excluded. Studies must include at least one arm of cash-only or plus-only treatment.

Condition or domain being studied

While social protection interventions have shown to improve health outcomes, the improvements often fail to sustain long-term (Roelen et al., 2017). As a result, these social protection interventions have begun to be packaged as cash-plus programmes, combining in-kind transfers with other interventions or services. These cash-plus interventions are argued to improve beyond cash alone because the ‘plus’ intervention specifically targets behavioural mediators that cash-alone does not change (Roelen et al., 2017).

These packaged interventions are at the forefront of the Nurturing Care Framework for Early Childhood Development, a recently launched joint initiative between UNICEF, World Health Organisation, the World Bank, and Partnership for Maternal, New-born & Child Health (ECD Action Network, 2018). This framework proposes an integrated response for young children across health, nutrition, child and social protection, and education sectors. However, despite the promise of these intervention packages in achieving multiple SDG targets for children, there has been no synthesis to evaluate if intervention packages across sectors are effective and if packaging the interventions have an interaction effect. Similarly, no synthesis has investigated whether these interventions reach the most vulnerable and close the equity gap (SDG 10) or how these interventions interact with the broader system.

Participants/population

1. The study must evaluate an intervention package implemented in one or more low- and middle-income countries, as defined by the World Bank.

2. The package must aim to improve outcomes in infants and children aged 0-5 years (0-59 months).

Intervention(s), exposure(s)

The intervention package must contain at least two components:

1) A cash transfer intervention (SDG1.3) that meets three requirements (adapted from Owusu-Addo and colleagues (2018)):

a. Includes financial assistance at the individual or household level;

b. Is non-contributory (i.e. individuals have not paid into the system) and in the form of a non-repayable, unrestricted grant (i.e. no requirement for how cash is used);

c. Aims to reduce the impacts of, or vulnerability to, poverty.

d. No restriction will be placed on behavioural conditions for receiving cash (i.e. both unconditional and conditional cash transfers will be included).

2) A ‘plus’ component targeting SDG2 (No Hunger); SDG3 (Good Health & Wellbeing); SDG4 (Education); SDG6 (Water & Sanitation), or SDG16 (Violence Prevention), specifically

a. Nutrition support programmes (including food transfers and behavioural change communication) to reduce malnutrition or food insecurity (SDG2.1-2.2);

b. Interventions to control communicable diseases and reduce infant/child mortality (SDG3.2-3.3);

c. Coverage from health insurance (SDG3.8);

d. Early childhood development, care and pre-primary education (SDG4.2);

e. Violence prevention and parenting interventions (SDG16.2).

3) These two components can either be a cross-sectoral linkage (a co-implemented intervention) or the ‘plus’ component can be nested within the cash transfer programme.

Comparator(s)/control

Receiving only one of the two interventions, treatment as usual, waitlist control, placebo (i.e. unrelated intervention), or no treatment. Studies without a control or valid counterfactual will be excluded. Because this review aims to evaluate potential interactions between cash and plus components, studies that evaluate no treatment (i.e. no cash or plus component) versus cash plus only will be excluded. Studies must include at least one arm of cash-only or plus-only treatment.

Main outcome(s)

Third-order SDG indicators related to children under five. These include measures of poverty (SDG 1.1.1-1.3.1); malnourishment (including stunting and obesity) (SDG 2.1.1-2.2.2); mortality (neonatal, infant, and children under 5) (SDG 3.2.1-3.2.2) including mortality from unsafe water or lack of sanitation/hygiene (SDG 3.9.2; 6.1.1-6.2.1) or infectious diseases (including HIV, tuberculosis, malaria, neglected tropical diseases) (SDG 3.3.1-3.3.5); being developmentally on track for health, learning and psychosocial wellbeing (SDG 4.2.1); participation in organised learning (SDG 4.2.2); physical punishment and/or psychological aggression by caregivers (SDG 16.2.1); birth registration (SDG 16.9.1).

* Measures of effect

No limits were placed on how outcomes were to be measured.

Additional outcome(s)

None

* Measures of effect

Not applicable

Data extraction (selection and coding)

Studies will be double screened and disagreements discussed. Authors will on the included studies. A second coder will also extract meta-analysis data, using a standardised extraction form.

Risk of bias (quality) assessment

Randomised studies will be assessed using the Cochrane Risk of Bias Tool and non-randomised studies using the ROBINS-I tool (Higgins et al., 2011; Sterne et al., 2016) and will be conducted blind by two independent researchers. Discrepancies will be discussed with a third author to reach consensus. The Cochrane Risk of Bias Tool assesses for bias of experimental studies related to: selection, performance, detection, attrition and reporting, among others (Higgins et al., 2011). The ROBINS-I tool evaluates bias at each phase of intervention (pre, during, and post) and to address the unique limitations of non-experimental studies (e.g. confounding) (Sterne et al., 2016). Both will be scored using + (low risk), ? (unsure), or – (high risk).

Strategy for data synthesis

It is anticipated that for some intervention packages (e.g. cash + nutrition support for stunting reduction), it may be possible to conduct a meta-analysis due to consistency in measures and number of studies. In cases of insufficient data for meta-analyses, a narrative synthesis approach will be used. The review will discuss the interventions according to the ten dimensions on intervention complexity and will note key themes as well as differences between the included interventions and any potential harms or unintended consequences.

The synthesis will also comment on the methodological underpinning of the evidence base. In particular, it aims to assess what study designs have been used to evaluate the packaged interventions, how long after

the intervention outcomes are evaluated, and if there is sufficient statistical power for assessing potential synergies between the interventions. It will also note if studies evaluate for potential spillover effects, the influence of context and heterogeneity effects and differential effects based on sex. Evaluating the influence of context helps understand the relation of the intervention to the broader system (e.g. humanitarian crises), if effects vary by context and the subsequent effect on changing health inequality rates, and considerations in bringing interventions to scale or new contexts.

Analysis of subgroups or subsets

Analyses will also investigate differential effects of multisectoral and nested cash-plus programmes as well as of unconditional and conditional cash transfers. Where there is sufficient data, sub-group analyses will be conducted on PROGRESS-PLUS criteria: place of residence; race/ethnicity; occupation; gender; religion; education; income; and social capital' (Evans & Brown, 2003).

Contact details for further information

Madison Little
madison.little@spi.ox.ac.uk

Organisational affiliation of the review

Centre for Evidence-Based Intervention, Department of Social Policy & Intervention, University of Oxford
https://www.spi.ox.ac.uk

Review team members and their organisational affiliations

Mr Madison Little. Centre for Evidence-Based Intervention, Department of Social Policy & Intervention, University of Oxford
Dr Keetie Roelen. Centre for Social Protection, Institute of Development Studies, University of Sussex
Dr Brittany CL Lange. Child Health & Development Institute of Connecticut
Dr Janina Steinert. School of Governance, Technical University of Munich
Dr Alexa Yakubovich. MAP Centre for Urban Solutions, Unity Health Toronto & University of Toronto
Professor Lucie Cluver. Centre for Evidence-Based Intervention, Department of Social Policy & Intervention, University of Oxford
Assistant/Associate Professor David K Humphreys. Centre for Evidence-Based Intervention, Department of Social Policy & Intervention, University of Oxford

Type and method of review

Intervention, Meta-analysis, Narrative synthesis, Systematic review

Anticipated or actual start date

01 September 2018

Anticipated completion date

31 January 2021

Funding sources/sponsors

This review is a part of Madison Little's DPhil in Social Intervention & Policy Evaluation and he is jointly funded by the ESRC Grand Union DTP, Clarendon Fund, and Green Templeton College, University of Oxford.

Conflicts of interest

Language

English

Country

Canada, England, Germany, United States of America

Stage of review

Review Ongoing

Subject index terms status

Subject indexing assigned by CRD

Subject index terms

Child; Conservation of Natural Resources; Financial Statements; Goals; Humans; Income; Infant

Date of registration in PROSPERO

10 September 2018

Date of first submission

10 September 2018

Stage of review at time of this submission

Stage	Started	Completed
Preliminary searches	Yes	Yes
Piloting of the study selection process	Yes	Yes
Formal screening of search results against eligibility criteria	Yes	Yes
Data extraction	Yes	Yes
Risk of bias (quality) assessment	Yes	Yes
Data analysis	Yes	Yes

Revision note

This revision adds a new author and updates the stage of the review. A two-year gap occurred in being able to work on this project, thus some minor adjustments to the protocol were made.

The record owner confirms that the information they have supplied for this submission is accurate and complete and they understand that deliberate provision of inaccurate information or omission of data may be construed as scientific misconduct.

The record owner confirms that they will update the status of the review when it is completed and will add publication details in due course.

Versions

10 September 2018
18 October 2018
22 December 2020

PROSPERO
This information has been provided by the named contact for this review. CRD has accepted this information in good faith and registered the review in PROSPERO. The registrant confirms that the information supplied for this submission is accurate and complete. CRD bears no responsibility or liability for the content of this registration record, any associated files or external websites.
