# Supplementary material for: Effectiveness of cash-plus programmes on early childhood outcomes compared to cash transfers alone: A systematic review and meta-analysis in low- and middle-income countries
Source: PLoS Med. 2021 Sep 28;18(9):e1003698. doi: 10.1371/journal.pmed.1003698 (PMC8478252; doi:10.1371/journal.pmed.1003698)
Supplement: Attachment — Submitted filename: little.pdf [file pmed.1003698.s008.pdf]

This paper describes a systematic review with meta–analysis of studies of cash–plus programmes in low and middle income countries.

I was asked for a statistical report and I interpret that to include all aspects of the design and conduct of the study.

Points of detail

Page 2 I know I am perhaps not the intended audience for this paper but the background section left me baffled as it assumes I know what a cash transfer programme is directed towards. I know now, obviously, having read the paper.

Page 2 It would be better to include confidence intervals for the effect sizes here, not just p–values.

Page 5 Claiming to be unique (also on page 25) is a bit of a hostage to fortune. There must be some form of words which still makes a claim for advancing the field without risk.

Page 8 In principle any machine readable article can be translated using on–line tools so I am not sure what is covered by 'a translation could not be obtained'. There has been at least one empirical study in this area (Jackson et al., 2019). So I think it might be better here to specify which languages the authors would have accepted. Later we ought to also know what articles fell through the net for language reasons.

Page 8 Was agreement between raters formally assessed?

Page 9 The statistical engine is R, RStudio is an IDE on top of it.

Page 9 Confidence intervals for I^2 would also be helpful to reveal our uncertainty about the heterogeneity. I know we did not use to use them but I am persuaded by Ioannidis et al. (2007) that we should. With relatively few studies here the intervals will be wide.

Page 15 We need to read on to page 17 to find out why Fenn and Levere are not in the forest plot. This could be flagged here.

Page 16 There must be considerable uncertainty about the exact value of I^2 here with small numbers of studies. I wonder whether it would be worth using the Knapp and Hartung adjustment here (Knapp and Hartung, 2003). It is available in the package the authors are using.

Page 17 Here we have some effect sizes which have lost their confidence intervals.

Supplement 1 This is one of the most comprehensive list of search targets I have ever seen.

Supplement 5 The authors do not make anything of these. I agree with their decision.

Point of more substance

I know that the modern view tends to the position that authors should report what was in the protocol, the whole protocol, and nothing but the protocol but is it wise to ignore studies of anaemia (page 30) just because the realisation that they existed came after the protocol was finished? I do not think readers would be justified in accusing the authors of going on a fishing expedition.

Summary

Mostly points for clarification.

Michael Dewey

References

J Ioannidis, N Patsopoulos, and E Evangelou. Uncertainty in heterogeneity estimates in meta–analyses. *British Medical Journal*, 335:914–916, 2007.

J L Jackson, A Kuriyama, A Anton, A Choi, J-P Fournier, A-K Geier, F Jacquerioz, D Kogan, and R Sun. The accuracy of Google Translate for abstracting data from non–English–language trials for systematic reviews. *Annals of Internal Medicine*, 171:677–679, 2019.

G Knapp and J Hartung. Improved tests for a random effects meta–regression with a single covariate. *Statistics in Medicine*, 22:2693–2710, 2003.
