# Supplementary material for: Effectiveness of cash-plus programmes on early childhood outcomes compared to cash transfers alone: A systematic review and meta-analysis in low- and middle-income countries
Source: PLoS Med. 2021 Sep 28;18(9):e1003698. doi: 10.1371/journal.pmed.1003698 (PMC8478252; doi:10.1371/journal.pmed.1003698)
Supplement: Attachment — Submitted filename: Response to Reviewers.docx [file pmed.1003698.s009.docx]

**Response to Reviewer Comments**

***Editor Comments:***

E1.01: Please revise your title according to PLOS Medicine's style. Your title must be nondeclarative and not a question. It should begin with main concept if possible. "Effect of" should be used only if causality can be inferred, i.e., for an RCT. Please place the study design ("A randomized controlled trial," "A retrospective study," "A modelling study," etc.) in the subtitle (ie, after a colon).

Change made (p.1 line 1-2). The new title is, “Effectiveness of cash-plus programmes on early childhood outcomes compared to cash transfers alone: A systematic review and meta-analysis in low- and middle-income countries.”

E1.02: Abstract: Please structure your abstract using the PLOS Medicine headings (Background, Methods and Findings, Conclusions; Please combine the Methods and Findings sections into one section, “Methods and findings”)

Change made.

E1.03 Abstract: Please provide the dates of search, types of study designs included, eligibility criteria including language of publication. Please briefly mention the countries represented in the studies, as well as some information on types of included studies, participants, and relevant characteristics of studies.

Changes made and details added: Search covers publications through 06 April 2021 (p.2 line 10), randomised and quasi-experimental studies (p.2 line 10-11), English publications (p.2 line 15). Countries represented and information on studies and populations (p.2 line 18-21): “Most interventions were implemented during the first 1000 days of the child’s life and were conducted in communities facing high rates of poverty and often, food insecurity. Evidence was found for ten LMICs, where most researchers used randomised, longitudinal study designs (n=14).”

E1.04 Abstract: Please remove italics used for emphasis.

Change made throughout abstract and manuscript.

E1.05 Abstract: As the final sentence of the Methods and Findings section, please describe the main limitation(s) of the study's methodology.

Change made (Abstract, p.3, Line 4-6). We added the sentence: “The main limitations of this review are the few numbers of studies that compared cash-plus interventions against cash alone and the potentially high heterogeneity between study findings.”

E1.06 Abstract: Conclusions: In the second sentence, we suggest phrasing this as the overall finding of your study, for example: “In this study, we observed that few package combinations showed evidence of effectiveness.” or similar.

Change made (Abstract, p.3 line 8-9). The sentence now reads: “In this study, we observed that few cash-plus combinations were more effective than cash transfers alone.”

E1.07 Author Summary: At this stage, we ask that you include a short, non-technical Author Summary of your research to make findings accessible to a wide audience that includes both scientists and non-scientists. The Author Summary should immediately follow the Abstract in your revised manuscript. This text is subject to editorial change and should be distinct from the scientific abstract.

Author summary added after abstract (p.3 line 14 to p.4 line 18).

E1.08 Throughout: Please use numbers within brackets for in-text reference citations, like this [1]. Please avoid the use of italics for emphasis.

Change made.

E1.09 Introduction: Page 5, Lines 9-10: Please temper this with “... to the best of our knowledge, to date there has been no synthesis to evaluate…” or similar.

Change made (Introduction p.6, Line 10-11). The sentence now reads: “However, to the best of our knowledge to date, there has been no synthesis to evaluate if these multisectoral interventions are more effective than cash transfers alone.”

E1.10 Methods: Page 5: Line 24: Please include the sentence "This study is reported as per the Preferred Reporting Items for Systematic Reviews and Meta-Analyses (PRISMA) guideline (S1_Checklist)."

Sentence added (Methodology p.6, line 26-27).

E1.11 Methods: Page 6, Lines 5-6: Please update your search to the present time.

The search has been updated to include studies through 06 April 2021 (noted in Methodology Information Sources & Search, p.7 line 6-7).

E1.12 Methods: Page 6-7: It would be preferable to format the description of eligibility criteria for included studies in paragraph form, rather than as an outline/list.

Change made, the outline form was transformed into paragraphs (Methodology Eligibility Criteria, p.7 line 12 to p.9 line 10).

E1.13 Methods: page 7, Lines 8-9: Please ensure that all abbreviations are fully spelled out at their first appearance in the main text (for example, UCTs, CCTs)

Abbreviations were corrected (noted in Methodology Eligibility Criteria p.8 line 9).

E1.14 Methods: Page 8: Please comment on assessment of publication bias.

Sentence added (Methodology Risk of Bias p.9 line 28 to p.10 line 2): “Due to a limited number of retrieved studies across a range of cash-plus combinations and outcomes, no meaningful analysis could be conducted to assess for publication bias.”

E1.15 Methods: Page 9, lines 9-10: Please clarify the description of correction for clustering- “The analysis corrected for clustering to address possible unit of analysis error”

Details added (Methodology Data Synthesis p.10 line 9-14): “The analysis corrected for clustering to address possible unit of analysis error by adjusting the sample size as necessary [24]. Specifically, in cases when studies allocated treatment at the cluster level but presented standard errors that were not adjusted for this clustering, the effective sample size was used in analysis. This value was calculated by dividing the original sample size by the design effect, which is computed using the average cluster size and intra-class correlation [25].”

E1.16 Methods: Page 10, line 6-7: “Eight protocols were identified that could meet the criteria for inclusion in an update to the review.” Was an attempt made to contact the authors to determine if the study findings have since been published?

Yes, we contacted all study teams, who all confirmed there was no available data or published findings. Sentences now read (Results Study Selection p.11 line 15-17): “Eleven protocols were identified that could meet the criteria for inclusion in an update to the review; study teams were contacted, who all confirmed no intervention impact data was yet available.”

E1.17 Results: Page 15, Line 7-8: Please present separate the results for the randomized vs. non-randomized studies, if possible.

This review only identified three non-randomised evaluations, of which two are on Cash + Primary Healthcare (the only studies for that intervention combination and, thus, discussed separately). The impact of study design impact from randomised/nonrandomised studies are discussed separately in the Risk of Bias section as well as collectively in the Recommendations for Research.

E1.18 Results: Throughout (for example page 23, line 19) please give the exact p value rather than p<0.05 for the meta-analysis results (however please report p<0.001 when appropriate).

Change made throughout manuscript.

E.19 References: Please use the "Vancouver" style for reference formatting, and see our website for other reference guidelines.

Change made throughout manuscript.

E1.20 PRISMA Checklist: Thank you for including the PRISMA checklist. Please update the checklist, using section and paragraph numbers to refer to locations within the text, instead of using page numbers.

The checklist has been updated (see Supplement 6).

E1.21 Figures 2, 3, 4: For the forest plots, please present the overall result from the meta-analysis, with 95% CI and p values.

All information is now included in the figures (Fig 2-7).

E1.22 Table 1: Please note in the legend that “CT” stands for cash transfer.

Change made (p.15).

***Reviewer #1 (Statistical Reviewer) Comments:***

R1.01 Page 2 I know I am perhaps not the intended audience for this paper, but the background section left me baffled as it assumes I know what a cash transfer programme is directed towards. I know now, obviously, having read the paper.

We have added additional information defining cash transfers (Introduction line 4-6): “Social protection interventions, including cash transfers (direct monetary provision), aim to mitigate the risk and effects of poverty and social exclusion [2]. Cash transfers often do not have restrictions on how the cash is spent, but may or may not have conditional requirements to receive the transfer (e.g., regular child growth monitoring).”

R1.02 Page 2 It would be better to include confidence intervals for the effect sizes here, not just p–values.

Confidence intervals are now included with all effect sizes throughout the abstract and manuscript.

R1.03 Page 5 Claiming to be unique (also on page 25) is a bit of a hostage to fortune. There must be some form of words which still makes a claim for advancing the field without risk.

Change made (Introduction p.6 line 9-11): “Cash-plus interventions hold much promise in accelerating achievement of multiple SDG targets for children. However, to the best of our knowledge to date, there has been no synthesis to evaluate if these multisectoral interventions are more effective than cash transfers alone.”

R1.04 Page 8 In principle any machine-readable article can be translated using on–line tools so I am not sure what is covered by ‘a translation could not be obtained’. There has been at least one empirical study in this area (Jackson et al., 2019). So I think it might be better here to specify which languages the authors would have accepted. Later we ought to also know what articles fell through the net for language reasons.

We clarified that the review only included English publications (Methodology Eligibility Criteria p.9 line 9). We discuss the one article that fell through (Discussion Limitations p.32 line 9-15): “Due to language abilities, this study only examined English studies; this may have also introduced selection bias through missing articles published through non-English outlets [61]. However, where known national programmes were operating or being planned, experts working on these programmes were contacted to identify potentially missed publications. Only one study was identified as being omitted by the search [62]. The cognitive impacts of this Cash + Psychosocial Stimulation intervention were published in English literature and included in this review [39]. However, the report also included nutritional impacts that were not reported in English.”

R1.05 Page 8 Was agreement between ratters formally assessed?

Yes, sentence with details on screening added (Methodology Study Selection & Data Extraction p.9 line14-15): “Initial agreement between raters was > 96% and reviewers agreed on the final included studies.” We also included assessment of rater agreement for the risk of bias (Methodology Risk of Bias p.9 line 25-26): “Piloting of the risk-of-bias assessment on six studies had 100% agreement between raters and > 95% for the full set of studies.”

R1.06 Page 9 The statistical engine is R, RStudio is an IDE on top of it.

Change made (Methodology Data Synthesis p.10 line 19).

R1.07 Page 9 Confidence intervals for I2 would also be helpful to reveal our uncertainty about the heterogeneity. I know we did not use to use them but I am persuaded by Ioannidis et al. (2007) that we should. With relatively few studies here the intervals will be wide.

Confidence intervals of I^2 were run for all meta-analyses and are included in-text.

R1.08 Page 15 We need to read on to page 17 to find out why Fenn and Levere are not in the forest plot. This could be flagged here.

These studies were dropped in response to Reviewer 3 comment 3.01.

R1.09 Page 16 There must be considerable uncertainty about the exact value of I2 here with small numbers of studies. I wonder whether it would be worth using the Knapp and Hartung adjustment here (Knapp and Hartung, 2003). It is available in the package the authors are using.

All meta-analyses were re-run using the new adjustment. The adjustment is noted in the Methodology Data Synthesis (p.10 line 19).

R1.10 Page 17 Here we have some effect sizes which have lost their confidence intervals.

While the particular paragraph referenced here was deleted, we have ensured that all effect sizes now have confidence intervals throughout the manuscript.

R1.11 Supplement 1 This is one of the most comprehensive list of search targets I have ever seen. Supplement 5 The authors do not make anything of these. I agree with their decision.

Thank you.

R1.12 I know that the modern view tends to the position that authors should report what was in the protocol, the whole protocol, and nothing but the protocol but is it wise to ignore studies of anaemia (page 30) just because the realisation that they existed came after the protocol was finished? I do not think readers would be justified in accusing the authors of going on a fishing expedition.

Anaemia data added (Results Cash + Nutrition BCC, p.20 line 16-17). (Due to some studies being removed in response to Reviewer 3 comment 3.01, there was only one study included in the review that discussed anaemia outcomes. Nonetheless, the data was added to the paper.)

***Reviewer #2 Comments***

R2.00 [Background] This paper explores an interesting topic by systematically examining results from published studies about cash transfers targeting infants or young children development outcomes. The originality of this study, compared to previous published reviews of the impact of cash transfers, is to address the delicate question of cash transfers combined with an additional intervention targeting child development and to explore whether this combination (named "Cash+") gives better results than one of the components alone (cash alone or "plus" alone). This kind of information is really needed to help decision makers, planners and various stakeholders design the best interventions possible in each particular context.

The study is rigorously conducted, the analysis includes meta-analyses when doable and the paper is well written. However, due to the small number of studies available, the variety of contexts and a large heterogeneity of the studies included in the review, both in terms of outcomes measured, in the kind of "plus" interventions and in the intervention designs, results are a bit disappointing. In the end, very limited evidence can be shown and no firm conclusion can be drawn from this work. The authors are not to be blamed for this, of course, and it's obvious that they did their best to assemble the available information, but the practical impact of such a study remains limited.

Thank you.

R2.01 One wonders if other choices could have been made to draw more information from the corpus of 63 articles that were assessed for eligibility, by applying less strict inclusion and/or exclusion criteria. It is out of the scope of the review of this paper to fully examine all 43 excluded articles, and I know that when undertaking such a systematic review the right balance is really difficult to establish between being very inclusive (and having to deal with more heterogeneity) or very exclusive (and ending up with a too few number of studies). Nevertheless, it seems to me that some criteria could have been less strict in order to increase the number of studies to be included, at least in the narrative analysis (the qualitative synthesis).

- For example, they considered as outcomes only what they call "third order SDG-indicators" related to children <5y old. The rationale behind this choice is not well explained and doesn't sound as the most effective choice. Indeed, when discussing their results, the authors themselves note that reaching "distant" outcomes is far more difficult than more proximal ones (discussion section, page 2 lines 10-18). Why not, then, having considered more proximal outcomes? Indeed, in this part of the discussion the authors could have elaborated a bit more about what implications does that have; in particular because many authors tend to think that there has been a too strong focus on ultimate (nutritional indices) outcomes in recent years, while many interventions achieve good results on more proximal outcomes. Also, to me the reference to SDGs is not very appropriate since SDGs target populations as a whole, not necessarily individuals (e.g. SDG targets 1.1.1 to 1.2.2, 2.1.1, 3.2.1, 3.2.2, 3.3.1-3.3.5… that the authors mentioned in their paper are all SDG indicators that are measured at the population level).

We are very appreciative of this thoughtful comment. We have incorporated this feedback in two ways. First, we added greater discussion to the study inclusion criteria (Eligibility criteria Outcomes, p.8 line 17-27) as to why we selected the outcomes for the study. (We also removed the SDG targets from the outcome descriptions.) Second, to address the scope of the review, the utility of the findings, and the interest in proximal outcomes, we have included data on the impact from cash-plus over cash alone on these intermediary outcomes within each cash-plus combination (drawn from the studies included in the review).

R2.02 - Also, the authors restrained the selection of studies to a list of "plus" components, referring again to the SDGs. First, in their protocol published on Prospero the authors mentioned SDG6 (water & sanitation) as one of the SDGs considered (Supplement 7, page 2) but they didn't mention that in the list of considered SDGs in the paper. If there was no study adding a WASH component to a cash transfer, which I doubt, they should at least have said so. Besides this, anyway, I think that referring to conceptual models of the young child development would have been a far better option than the SDGs (that don't have any conceptual model behind; and are not specifically targeting the child development per se).

We added clarification about water and sanitation interventions under the study inclusion criteria (Methodology Eligibility criteria, p.8 Line 5-7). In terms of the conceptual framework of the review, we had used the SDG framework in designing the study and drawn the framework from literature that conducted network analyses to demonstrate that the SDGs are inter-related (e.g., Nunes et al 2016 – mentioned in introduction). Nonetheless, we agree with your point that it is useful to link to child development frameworks and we have linked our findings to this latter framework in the discussion (Discussion Summary of evidence, p.29 Line 1-4).: “These intervention packages also touch all five areas of the multisectoral Nurturing Care Framework for Early Child Development, namely by integrating cash (Security & safety) with efforts to drive 1) Adequate nutrition, 2) Good health, 3) Opportunities for early learning, and 4) Responsive caregiving [8].”

R2.03 - Still in terms of studies selection, the authors could also have thought back of the final objective of their work, which might have been to give useful information for planners rather than highlighting a lack of evidence on a narrower question: indeed, by strictly focusing on interactions between cash and plus components they excluded studies with a 'cash-alone' and a 'cash+' arms, but without a 'plus alone' arm. At the end of the day, this is likely to have prevented them to examine more in-depth the impact of cash+ versus cash-alone interventions, which is a very interesting question. Interestingly enough, the first sentence in the conclusions says "Questions in social protection have remained as to what complementary interventions are necessary for cash transfers to have maximal impact" (page 31, lines 1-2). Which is exactly my point, but not exactly the question they addressed in their review.

To clarify, the initial manuscript did include studies that had cash-only and cash-plus arms but no plus-only arm. However, as also mentioned by Reviewer 3 (comment 3.01), the message was a bit muddled by including studies that either evaluated cash-plus vs cash-only or cash-plus vs plus-only. Thus, we have restricted the review to studies that have cash-only and cash-plus arms, which allows us to concentrate on whether cash-plus interventions are more effective than cash transfers alone. We believe that these changes strengthen the message of the review and the clarity of our findings.

R2.04 - I also checked one or two excluded studies and failed to find an obvious reason for exclusion, even with the strict criteria applied in the study (e.g. the first in the alphabetical order: Adubra et al. 2019). Again, I know how harsh it is to conduct such a systematic analysis. All the above is not to say that the study was badly conducted (which is not the case) but I wanted to highlight that other choices could have led to a more informative review. This can't be for sure, of course, but this deserves at least to be discussed quite extensively in the discussion section.

We clarified the reasons for full-text exclusion for each individual article rather than an aggregated list as in the initial manuscript (new change in Supplement 2). As noted in our comments above, we have made the review more informative by discussing the impact of cash-plus above cash-alone on the causal pathways (and on proximal outcomes) throughout the results and in the discussion section.

R2.05 Another major point I'd like to make regards the absence of information about the evaluation designs. While there is information about duration, age range, etc., it's obvious when reading the abstracts of the included studies that some of them used longitudinal designs (same children are followed-up and surveyed at baseline and endline) while others are based on repeated cross-sectional surveys at baseline and endline, on children of the same age range. This makes a huge difference in terms of interpretation, potential biases, duration of exposition to the intervention (for repeated cross-sectional designs) and statistical power. I found it very strange that this was not accounted for in the study and that this point is not even mentioned I the discussion section.

We added greater details about the study designs and its impact on findings in the Risk of Bias section (see Results, Risk of Bias, p.17 Line 5-10) and added more about the role of study designs in the Discussion Recommendation for Research (p. 32, line 18 to p.33 line 8).

R2.06 Also, regarding studies for which the outcome is the child undernutrition, the authors analysed the impact in terms of rates of undernutrition. This is not the most powerful analysis, in particular for studies using a longitudinal evaluation design. Why not analysing the results in terms of changes of the continuous underlying variables (e.g. height-for-age or weight-for-length/height z-scores)?

We have also included meta-analyses now on z-scores for undernutrition (Results Synthesis of Results Cash + BCC p.17 line 27 to p.18 line 2, Figure 3 p.19). We have also included new meta-analyses on Cash + Food and have included z-score meta-analyses there as well (Results Synthesis of Results Cash + Food p.21 line 19-26).

R2.07 My last major comment regards the discussion section. The paper is already quite long; results are well and quite extensively described and the discussion starts with a summary of the results (page 25), which is good. As said above, and as acknowledged by the authors, at this point we don't have much evidence, no firm conclusions, nor much information we don't already know. I understand that the authors made some efforts to comment about some characteristics of cash or cash+ interventions that could increase impacts on some outcomes. But let's face it: they don't have much to say that really comes from their study.

The discussion section has been re-worked in light of the revised findings as well as including additional information on the proximal outcomes within the studies. We believe the section has been strengthened and any peripheral commentary removed.

R2.08 - About the context, for example, they draw some comments on the role of food transfers (page 26, first paragraph) but all what that says is that in emergency contexts, when dealing with acute undernutrition in infants and young children, it is key to give access to some nutritious foods (that are indeed most often ready-to-use supplementary foods) on top of the cash transfer. We already know that but it's good to have some combined evidence. However, the rest of the paragraph is made of more general statements that are not really related to the work presented.

Paragraph deleted.

R2.09 - Similarly, when commenting on the importance of the age of children for interventions targeting stunting (page 26, lines 14-18), the authors refer to the "wider literature in cash transfers" while in fact this has been demonstrated years ago in the nutrition literature. And in a few other parts of the discussion, the authors err on the side of making general comments that are not directly related to their results. This is likely because results are poor, of course, but then the discussion can be shorter.

Paragraph deleted and discussion tightened.

R2.10 - The first paragraph of the recommendations for research, again, describes good but already well known points about cash or + intervention components, but those statements don't come specifically from this review.

Paragraph deleted.

R2.11 - Please avoid the term "malnutrition" when it obviously refers to "undernutrition" (which is the preferred term, since "malnutrition" encompasses also overweight, obesity and related noncommunicable diseases). Page 7 line 18, on the contrary, "malnutrition" should replace "malnourishment".

Change made throughout the manuscript.

R2.12 - Please note also that "underweight" is not a type of undernutrition (such as stunting and wasting); it is, indeed, only an anthropometric indicator of one of the two types above, or both.

Change made throughout manuscript.

R2.13 UCT and CCT acronyms are used without being defined

Abbreviations were corrected (noted in Methodology Eligibility Criteria p.8 line 9).

R2.14 Page 10, lines 8-9: this last sentence of the paragraph belongs to the methods section (where it is already said: page 9, line 5) but not to the results section.

Duplicate sentence deleted from results.

R2.15 Page 11, lines 3-4: "CCTs (7 studies), UCTs (11 studies), and public works programmes (3 studies)" add up to 21 studies, not 20.

Numbers were corrected (Results Study Characteristics p.12 line 6-7). The studies now add up to the number of included studies in the review (17): “CCTs (6 studies), UCTs (7 studies), and public works programmes (4 studies) were studied.”

R2.16 Page 11, lines 7-9: even if the information is in the table, it would be good to have the number of studies for each category of 'plus' components.

Numbers were added (Results Study Characteristics p.12 line 8-11).

***Reviewer #3 Comments:***

R3.00 [Background] This is a timely and important topic, as more countries and development partners increasingly implement cash plus or integrated programming to address multidimensional poverty. This paper is well motivated and the background is well written.

Thank you.

R3.01 I find the comparison to either cash alone or plus alone somewhat problematic. If the authors stick to cash plus v. cash only, then you can make conclusions about whether 1) the plus component is effective or 2) "cash plus" is more effective that cash alone (if there are no differences you would conclude it is not). However, the combination of comparing cash plus to either cash alone or plus alone makes it difficult to understand what the conclusion is. I agree with their decision to do meta-analysis on cash plus v. cash only (avoiding mixing in of plus only with cash only as a comparison) but question the descriptive inclusion of cash plus to plus only. In an ideal world, you would want to compare studies that had the following three arms: 1) cash only, 2) plus only, 3) cash plus and 4) controls. This would allow you to understand impacts of "cash plus" and the synergies of cash and plus components (above and beyond either component separately). However, the authors
could not do this because a sufficient number of studies with this design does not exist. Thus, the authors had to make a choice and in the background/framing, they seem to choose not to understand overall impacts of cash plus, but rather they start with cash as "standard of care" and seek to understand whether cash plus is more effective than cash alone. Following from this logic and the theoretical framing, they should drop the comparison to plus only, which just tells you whether cash is effective by itself or whether the combination is more effective than services/other interventions alone.

This has been a very useful comment. We removed the studies that evaluated only plus-alone vs cash-plus and focused the paper on the effectiveness of cash-plus compared to cash alone. We also incorporate the comment on ideal study design into the Recommendations for Research (p.32 line 18 to p.33 line 8).

R3.02 Page 21: Lines 6-11: It's not clear whether the discussion of heterogenous impacts follows the current paper's model of cash plus v. cash only, or whether they are citing findings from the original studies being reviewed, which may have compared to a pure control, and therefore should be removed from the results section.

Language was clarified (Results Cash + Psychosocial Stimulation, p.26 line 4-9). Paragraph now reads: “Comparing different socioeconomic subgroups in cash-plus arms to cash alone, these intervention packages varied in effects. While there was evidence of higher cognitive impact for Mexican indigenous communities in the stratified sample and among children with lowest scores at baseline, the intervention effects had a strong benefit to wealthier households and no benefit to the lowest income households [39]. Two studies in Colombia and Mexico found the interventions favoured mothers with greater education [39,50].”

R3.03 This line seems out of place and doesn't connect to the sentence or larger paper: "one study emphasises that the burden of high fertility rates puts pressure on already strained social services in meeting the demands for early child development (Premand & Barry, 2020)."

Sentence deleted.

R3.04 The language on page 26, line 27 should be tempered. While the studies examined are rigorous, there are still a very limited number of studies examining cash plus v. cash alone and therefore the ability to generalize is still limited until more studies are published.

Change made (Discussion Summary of Evidence p.29 line 20-22): “Overall, the studies included in this review had low risk of bias and while the studies were rigorously conducted, there are still a very limited number of studies examining cash-plus versus cash alone and therefore, the ability to generalise is limited until more studies are published.”

R3.05 In the comparison to Manley et al. (2020), can the authors add a sentence to explicitly state how this study adds to the literature (i.e., what the combined findings suggest)? They appear to be saying that since Manley et al. found impacts of cash plus or cash plus on stunting, and the current authors find no difference between cash only and cash plus, that cash alone appears to be driving the impacts. It would help if the authors could clarify their conclusion.

We clarified our conclusion, namely that any impacts were likely due to the cash component (Discussion Summary of Evidence p.30 line 14-25). “Our review adds to the evidence base by specifically comparing Cash + Nutrition BCC to cash alone, finding that there was no statistically significant difference between the two interventions in reducing stunting; this may suggest that any improvements in stunting reduction are likely driven by the cash component rather than the added Nutrition BCC.”

R3.06 This conclusion should be tempered and contexualized within the limitations of the study: "Findings also suggest limited or no added impact of nutrition behaviour change and psychosocial stimulation interventions and possibly worsening inequality from some cash-plus combinations compared to cash alone." In fact, Roy et al. (2019) have found that in terms of sustained impacts in Bangladesh, transfers + BCC had sustained impacts whereas transfers only did not on several pathways and outcomes important for child health (i.e., maternal IPV, economic resources, agency, social control and household poverty). The limitations of the current paper should be more clearly stated, that they are simply comparing effectiveness of the "plus" components and in fact have not compared cash v. control as compared to cash plus v. control. The statements/conclusions they are making would require the latter, which the authors have not done.

Sentence deleted.
